# Mapping functional traces of opioid memories in the rat brain

**DOI:** 10.1093/braincomms/fcae281

**Published:** 2024-08-19

**Authors:** Joana Gomes-Ribeiro, João Martins, José Sereno, Samuel Deslauriers-Gauthier, Teresa Summavielle, Joana E Coelho, Miguel Remondes, Miguel Castelo-Branco, Luísa V Lopes

**Affiliations:** Instituto de Medicina Molecular João Lobo Antunes, Faculdade de Medicina de Lisboa, Universidade de Lisboa, 1649-028 Lisboa, Portugal; Coimbra Institute for Biomedical Imaging and Translational Research (CIBIT), Institute for Nuclear Sciences Applied to Health (ICNAS), University of Coimbra, 3000-548 Coimbra, Portugal; Coimbra Institute for Biomedical Imaging and Translational Research (CIBIT), Institute for Nuclear Sciences Applied to Health (ICNAS), University of Coimbra, 3000-548 Coimbra, Portugal; CQC, Chemistry Department, University of Coimbra, 3004-535 Coimbra, Portugal; Centre Inria d'Université Côte d'Azur, 06902 Valbonne, France; Addiction Biology Group, i3S- Instituto de Investigação e Inovação em Saúde (i3S), Universidade do Porto, 4200-135 Porto, Portugal; ESS, Polytechnic of Porto, 4200-072 Porto, Portugal; Instituto de Medicina Molecular João Lobo Antunes, Faculdade de Medicina de Lisboa, Universidade de Lisboa, 1649-028 Lisboa, Portugal; Instituto de Medicina Molecular João Lobo Antunes, Faculdade de Medicina de Lisboa, Universidade de Lisboa, 1649-028 Lisboa, Portugal; Faculdade de Medicina Veterinária, Universidade Lusófona, 1749-024 Lisboa, Portugal; Coimbra Institute for Biomedical Imaging and Translational Research (CIBIT), Institute for Nuclear Sciences Applied to Health (ICNAS), University of Coimbra, 3000-548 Coimbra, Portugal; Faculty of Medicine, University of Coimbra, 3000-370 Coimbra, Portugal; Instituto de Medicina Molecular João Lobo Antunes, Faculdade de Medicina de Lisboa, Universidade de Lisboa, 1649-028 Lisboa, Portugal

**Keywords:** functional connectivity, whole-brain activity, conditioned place preference, fMRI, rat brain

## Abstract

Addiction to psychoactive substances is a maladaptive learned behaviour. Contexts surrounding drug use integrate this aberrant mnemonic process and hold strong relapse-triggering ability. Here, we asked where context and salience might be concurrently represented in the brain during retrieval of drug–context paired associations. For this, we developed a morphine-conditioned place preference protocol that allows contextual stimuli presentation inside a magnetic resonance imaging scanner and investigated differences in activity and connectivity at context recall. We found context-specific responses to stimulus onset in multiple brain regions, namely, limbic, sensory and striatal. Differences in functional interconnectivity were found among amygdala, lateral habenula, and lateral septum. We also investigated alterations to resting-state functional connectivity and found increased centrality of the lateral septum in a proposed limbic network, as well as increased functional connectivity of the lateral habenula and hippocampal ‘cornu ammonis’ 1 region, after a protocol of associative drug–context. Finally, we found that pre- conditioned place preference resting-state connectivity of the lateral habenula and amygdala was predictive of inter-individual conditioned place preference score differences. Overall, our findings show that drug and saline-paired contexts establish distinct memory traces in overlapping functional brain microcircuits and that intrinsic connectivity of the habenula, septum, and amygdala likely underlies the individual maladaptive contextual learning to opioid exposure. We have identified functional maps of acquisition and retrieval of drug-related memory that may support the relapse-triggering ability of opioid-associated sensory and contextual cues. These findings may clarify the inter-individual sensitivity and vulnerability seen in addiction to opioids found in humans.

## Teaser

Gomes-Ribeiro *et al*. have developed a rodent morphine-conditioning protocol whose contextual cues are adapted to functional MRI. They found that animals exhibiting stronger emotional responses develop stronger connectivity patterns, anticipating stronger morphine addiction behaviour. These brain patterns may explain the inter-individual sensitivity and why some individuals are more vulnerable to addiction to opioids.

## Graphical Abstract

**Graphical Abstract:**
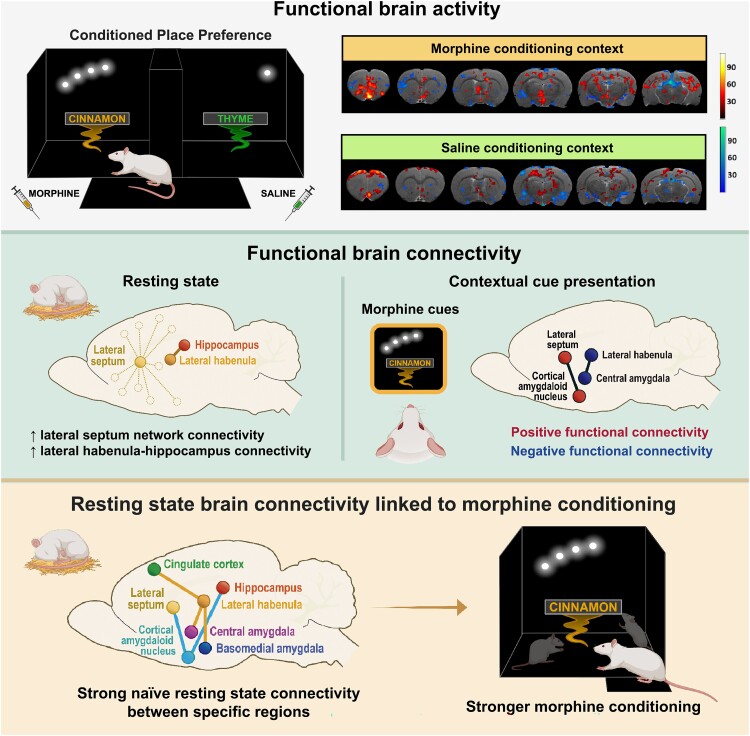

## Introduction

Opioids are classical central nervous system depressants, the most effective anti-nociceptive drugs, that create strong physical and emotional dependence, frequently leading to overdose.^1^ Opioids bind to opioid receptors in local and efferent GABAergic neurons of the ventral tegmental area (VTA), leading to their hyperpolarization and silencing, in turn allowing dopaminergic (DA) neurons to switch from tonic to phasic firing modes.^2^ The consequent surge of dopamine release onto target regions of the meso-cortico-limbic system^3^ causes an intense sense of euphoria, pleasure, and relaxation.^4,5^ Such positive affect constitutes a strongly salient stimulus, driving continued opioid use and triggering a set of molecular and physiological changes in the brain, including reduced opioid receptor availability,^6^ synaptic plasticity in VTA neuronal populations^7^ and altered neurotransmission.^8,9^ Such homeostatic buffering becomes ineffective in the absence of the drug, leading to activation of stress systems, such as the k-opioid receptor/dynorphin system and the increased release of corticotropin-releasing hormone (CRH),^10,11^ causing a severe withdrawal syndrome (e.g. dysphoria, pain, anxiety and depression), and dependence.^12^ These aversive physical and emotional states, stronger than those found with other types of drugs (e.g. amphetamine and cocaine),^13^ can only be avoided, or relieved, by retaking the drug, further reinforcing the motivational value of opioids.^14,15^ Thus, drug addiction is a maladaptive learning process.

Drug use becomes strongly associated with surrounding context, and emotional states become conditioned to the perception of environmental stimuli.^14-16^ Contextual cues such as substance use paraphernalia, visual scenery, odours and sounds, are encoded in the brain^17^ and become motivationally charged in strong associative memories.^18^ Once present, such stimuli can lead to the reactivation of these stable memories and on their own drive opioid (re)use, even after extinction.^19^ Thus, context plays a critical role in repeated drug use and relapse. In spite of intense research, the neural mechanisms responsible for maintaining and retrieving these maladaptive memories are still a matter of discussion.

The conditioned place preference (CPP) paradigm is commonly used to model drug–context associations, by which the positive affect resulting from opioid use becomes conditioned to sensory cues present in an enclosed environment.^20^ In natural environments, contextual information is initially represented in the hippocampus (HIPP) before being transferred to higher-order cortical centres for long-term memory storage.^21^ The strong reciprocal HIPP–cortical connections, along with its numerous subcortical inputs and outputs, place the HIPP as a hub for encoding and retrieval of drug–context memories, something that has been shown in early HIPP lesion studies, with hippocampectomized rats unable of acquiring drug-induced PP.^22^ Consistent with this, morphine-CPP retrieval was associated with increased basal synaptic transmission and impaired long-term potentiation in the HIPP.^23^ In addition, increased activity-related c-Fos expression was detected in dentate gyrus (DG) granule cells, involved in context discrimination,^24^ upon exposure to a morphine-related context.^25^ Finally, reward expectancy in the morphine-CPP paradigm increased high-frequency gamma oscillations in the ventral HIPP–NAc circuit.^26^

Despite numerous reports highlighting the representation of drug-associated contexts in the HIPP, recent contextual fear-conditioning studies show that memory engrams are formed, and consolidated, in the HIPP and other connected structures such as mPFC and basolateral amygdala (BLA).^27^ Such remote memory encoding is thought to support the stabilization of HIPP representations of contexts before they gradually mature in the PFC.^28^ Given the considerable midbrain dopaminergic innervation of the HIPP, and its role in stabilizing neural maps biased towards particularly rewarding events,^29,30^ VTA is another region likely playing a role in the regulation of encoding and reactivation of ‘drug maps’.

To identify the neural circuits underlying drug-induced memory storage and retrieval, we started with a classical morphine-CPP apparatus, and redesigned the conditioning cues allowing the contextual stimuli to be presented inside a rodent MRI apparatus. This setup allowed us to test the hypothesis that the inter-individual heterogeneity of CPP score could be linked to differences in functional connectivity in memory-related and/or valence-assignment regions during associative morphine- and saline-paired contexts.

## Materials and methods

### Subjects

Animal procedures were performed at the Rodent Facility of Instituto de Medicina Molecular (iMM), licensed under the reference number 017918/2021, and at Institute for Nuclear Sciences Applied to Health (ICNAS) Pre-Clinical Facility, in compliance with the European Directive 2010/63/EU, transposed to Portuguese legislation in DL 133/2013. All animal research projects were reviewed by the Animal Welfare Bodies (ORBEA) of each institution and approved by national authority, to ensure animal use was in accordance with the applicable legislation and following the 3Rs principle. Male Sprague–Dawley rats (Charles River Laboratories, France) aged 12–16 weeks were used for all behavioural experiments (total *n* = 30). In this study, we followed a well-established morphine-CPP protocol^31^ described in male rats, as reference for our protocol implementation into functional MRI (fMRI), and therefore, male rats were used. Environmental conditions were kept constant: food and water *ad libitum*, 22–24°C, 45–65% relative humidity, 12 h light/dark cycles and housed in groups of five, except for the MRI group which was housed in pairs. At this age, we have not found any evidence of the influence of housing arrangements (two to five animals per cage) in memory-related behaviour, in rats.^32^

### Drugs

Rats were administered either a morphine hydrochloride (5 mg/kg SC) or saline (0.9%) solution. For fMRI experiments, an induction dose of isoflurane (2–3%) and both bolus (0.05 mg/kg) and maintenance (0.1 mg/kg/hr) doses of the α_2_ adrenergic agonist Medetomidine were administered for light sedation. This is proven to be efficient in immobilizing the animal inside the MRI scanner for several hours, all the while maintaining neural activity and neurovascular coupling intact.^33^ Plus, a previous study showed that α_2_ adrenergic activation with another agonist, clonidine, does not affect the discriminative stimulus effects of morphine in rats.^34^ All procedures were performed in accordance with EU and Institutional guidelines.

### Behavioural experiments

#### Conditioned place preference adaptation

We aimed to develop a conditioning paradigm that could be effectively transferred to an MRI scanner. Selection of conditioning cues started with an assessment of spontaneous odour preference for a variety of odorants, as described in Lee *et al*.^35^ Male Sprague–Dawley rats (*n* = 8) were isolated in a common rat cage and presented with herb and/or spice water infusions. A few millilitres was applied onto a filter paper in an inverted small petri dish placed on top of the cage grid. We then timed the active investigation, defined as periods in which the animal's nose was within 1 cm of the petri dish, during a 120 s time lapse. Rats completed six trials in the 1st day and seven on the 2nd day. Odourant presentation sequence was randomized across rats at 2 min intervals. We then normalized investigation times by dividing the time spent in the vicinity of an odourant, and the summed investigation times of all odourants on that day. This number was then divided by the water exploration time to get a normalized preference metric. Normalized preference was then compared across odourants using ANOVA followed by *post hoc* comparisons (Fisher LSD). This study showed that basil and bay were the least ‘preferred’ odourants. All other odourants were considered ‘neutral’ for the purpose of this research (Supplementary Fig. 1A).

The two odourants eliciting similar investigation times (cinnamon and thyme) were then transferred to the CPP behavioural apparatus. This consisted of a black Plexiglas box divided into three compartments: two main squared chambers of 40 × 40 × 40 cm, each opened by a 15 × 40 cm entrance, each containing one or the other odour cues, connected by an external neutral compartment of 40 × 15 × 40 cm. The animals were placed in the neutral compartment of the CPP apparatus, and the exploration times of the conditioning chambers were assessed for 15 min. Rats showed no preference for either context as exploration times were similar (Supplementary Fig. 1C). Next, we coupled each of the two presented odorants with visual cues composed of either one or a set of four aligned equidistant white LEDs at minimal intensity, which were placed above eye level on three out of the four black walls of the conditioning chambers. The rat's visual system mainly allows the detection of light contrasts and intensities and less individual colours.^36^ The single and four-set LED visual stimuli were paired, respectively, with thyme- and cinnamon-odourized black foam sheets located near the base of the compartment walls. No additional contextual cues were present (e.g. neither the floors nor the walls contained any textures).

#### Morphine-conditioned place preference protocol

**Figure 1:**
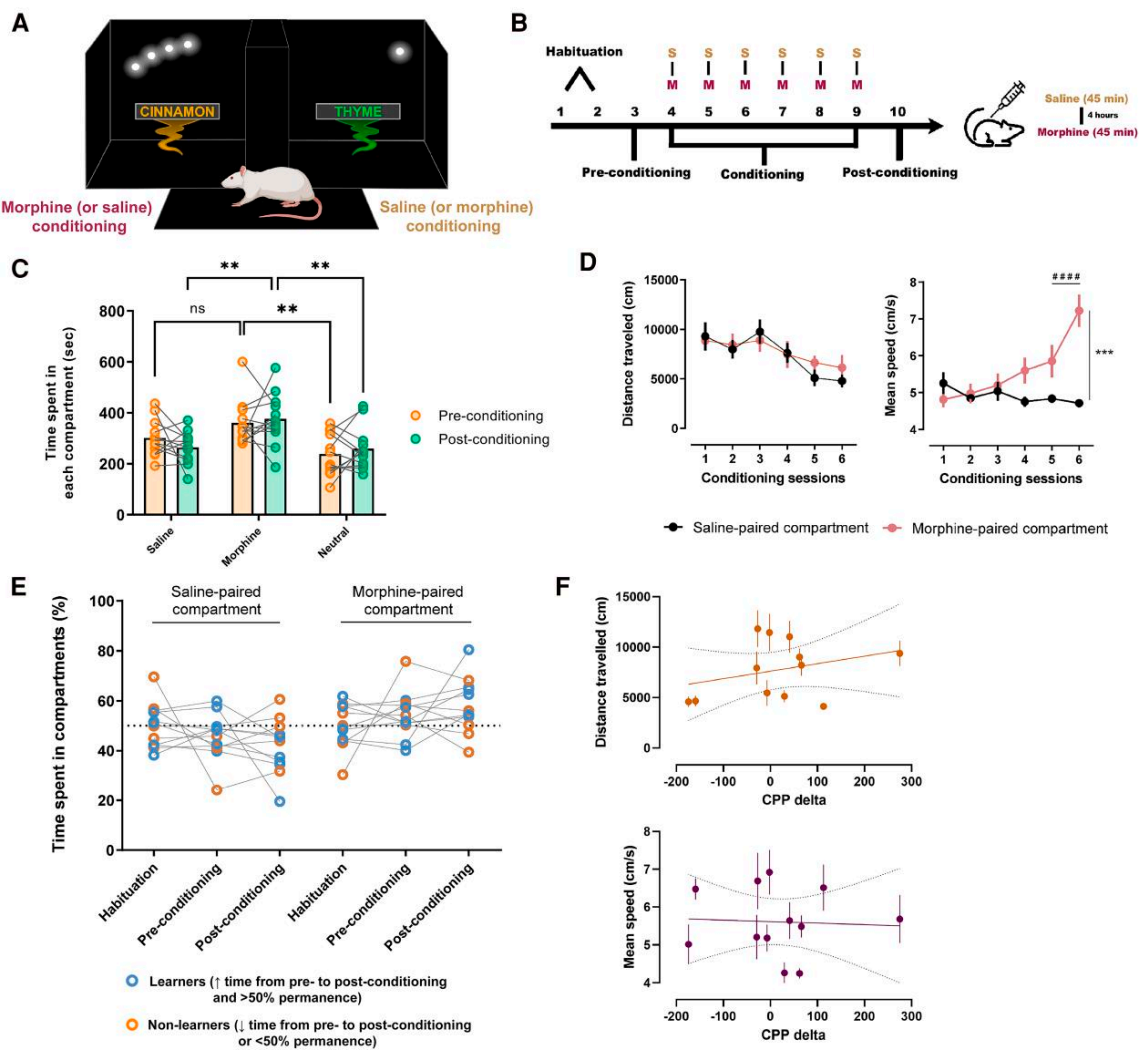
**Conception of elementary and MRI-adaptable conditioned place preference contexts for functional brain imaging.** (**A**) Outline of the CPP conditioning chambers upon implementation of simple contextual cues. During free exploration of the conditioning apparatus, rats could find either a cinnamon-odourized chamber paired with small lines of white light on a dark background, or a thyme-odourized chamber illuminated by a single point of light. The unconditioned stimulus (morphine) was delivered in a counterbalanced and unbiased fashion. (**B**) Timeline for the CPP protocol. Rats were allowed free exploration of the apparatus for 2 days (habituation and pre-conditioning), and on conditioning days, they received a subcutaneous injection of saline before being placed in one of the chambers. Four hours later, animals were administered morphine and placed in the opposite chamber. The procedure was repeated for 6six days. One day after the last conditioning session, animals were tested for place preference. (**C**) Rats developed preference for the morphine-paired context, as revealed by the time spent in the morphine-paired compartment relative to time spent in the saline-paired compartment at post-conditioning. A two-way repeated measures ANOVA followed by Bonferroni's multiple comparisons test was employed to analyse differences between times spent in the two chambers. Values are presented as mean ± SEM. **P < 0.01. *n* = 12 rats. (**D**) Locomotion parameters such as distance travelled (cm) and mean speed (cm/s) were measured across conditioning sessions in both compartments. Animals showed a gradual increase in mean speed throughout conditioning in the morphine-paired compartments, while maintaining stable walking distances (two-way repeated measures ANOVA followed by Bonferroni's multiple comparisons test). Values are presented as mean ± SEM. ^####^*P* < 0.0001; ***P = 0.0006. *n* = 12 rats. (**E**) Individual permanence times in the two CPP compartments shows that M-CPP was expressed in 7 out of 12 animals. Clusters of learners and non-learners were defined based on two factors: direction of change of time spent in the compartments from pre- to post-conditioning and percentage limit (if time increased and was over 50%, learners; if time decreased or was below 50%, non-learners). *n* = 12 rats. (**F**) Based on the difference between time spent in the morphine-paired compartment post- versus pre-conditioning (in s), each animal was attributed a CPP-delta value. he plots show the correlation between these delta values and the two locomotion parameters. To calculate this regression, we used the mean of the six conditioning days for both distance travelled and mean speed, for all animals (simple linear regression, distance travelled, *R*^2^ = 0.094; *F*_(1,10)_ = 1.041, *P* = 0.332; mean speed, *R*^2^ = 0.0028; *F*_(1,10)_ = 0.028, *P* = 0.871). *n* = 12 rats.

Male Sprague–Dawley rats (12 weeks old, *n* = 12) were conditioned in a counterbalanced fashion, according to Fig. 1A. Rats were allowed free exploration of the apparatus for 2 days (habituation and pre-conditioning) during 15 min, and on conditioning days, they received a subcutaneous injection of saline before being placed in one of the chambers for 45 min. Four hours later, animals were administered morphine (5 mg/kg SC; as in Wu *et al*.^31^) and placed in the opposite chamber for 45 min. The procedure was repeated for 6six days, at the same time of day for each animal. The assignment of conditioning compartments for each rat was randomized and unbiased, counterbalanced for each compartment/cue pair, since we found no differences in the initial individual preferences (as measured by time spent in each compartment at pre-conditioning). One day after the last conditioning session, animals were tested for PP while freely exploring the apparatus for 15 min. To measure the effectiveness of conditioning, we compared the time spent in the morphine-paired compartment by calculating the difference at post-conditioning versus pre-conditioning (CPP-delta). Rats showing both an increased time spent in the morphine-paired compartment from pre- to post-conditioning and >50% permanence therein were classified as ‘learners’. Rats showing both a decrease in time spent in the morphine-paired compartment from pre- to post-conditioning and <50% permanence therein were classified as ‘non-learners’. Behavioural metrics such as time spent in compartments, distance travelled and mean speed (without rest) were extracted from SMART video tracking system (v.3.0, Panlab, Spain). To assess differences between permanence times in the saline and morphine compartments, we used a two-way repeated measures ANOVA, followed by *post hoc* comparisons using Bonferroni's correction. Values for permanence in saline- or morphine-paired compartments are reported as time (s) ± SEM. This protocol was validated in an independent experiment where a group of animals showed a strong preference for the drug-paired context (Supplementary Fig. 1C).

### MRI experiments

**Figure 2:**
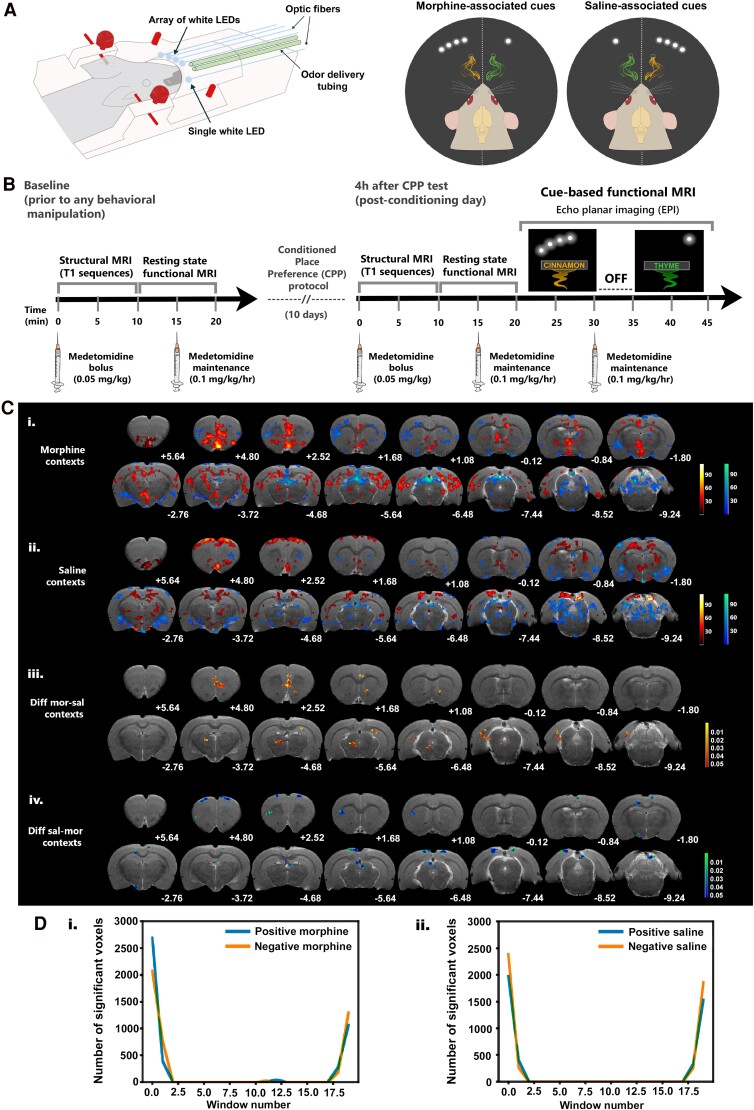
**Responses to saline- and morphine-associated cues show regional segregation in the rat brain and are (re)activated early on after onset of contextual cue presentation.** (**A**) Scheme of replication of conditioning contexts in the MRI scanner. Optic fibres connected to white LEDs and air-puff tubes connected to an olfactometer were placed around the MRI stereotactic frame (left, side view; right, front view). Stimuli were presented in a counterbalanced fashion, mimicking the contexts created in the behavioural apparatus. Thus, for six animals, the morphine-paired context was cued by the array of LEDs combined with the scent of cinnamon, and the saline-paired context was cued by the single point of light combined with the scent of thyme. For the other six animals, context assignment was reversed. (**B)** Timelines depicting our functional whole-brain imaging protocol. For all fMRI experiments, sedation was maintained by α_2_-adrenergic agonist medetomidine (intraperitoneal bolus at 0.05 mg/kg; subcutaneous maintenance infusion at a rate of 0.1 mg/kg/h, every 15 min after bolus injection). Animals were imaged at two time points: before and after behavioural manipulation in M-CPP. Baseline resting-state fMRI was acquired before initiation of behavioural testing (left timeline); post-CPP resting-state fMRI was acquired ∼2 weeks after the first set of baseline acquisitions and 4 h after the M-CPP post-conditioning (or test; right timeline). Anatomical scans were acquired prior to resting-state fMRI. Cue-based fMRI was acquired in two 10 min blocks, and the pairs of contextual cues were presented in the same order for all animals, regardless of whether they were previously associated with morphine or saline administration in the CPP box. The two stimuli were separated by a 5 min inter-block interval. (**C**) Representative anatomical scans (T_2_-weighted anatomical MRI image of our template brain) display: i. and ii. Mean BOLD activity maps within the first 30 s window in response to the morphine- and saline-associated cues, respectively. Colour scale represents mean regression coefficients for morphine and saline contrasts in comparison to baseline (masked under a significance level set at 0.05 and a cluster size threshold of 16 voxels); and iii. and iv. Magnitude maps show the clusters that survived the difference between significantly activated clusters during presentation of saline and morphine cues, and *vice versa*. Colour scale represents *P* values of the surviving voxels. Coordinates under each brain section represent Paxinos and Watson's^37^ rat brain atlas coordinates (in mm). *n* = 12 rats. (**D**) Representation of the number of activated voxels in the whole brain across the 10 min context presentation sequence for the i. morphine and ii. saline contexts. *n* = 12 rats.

We replicated the odour–visual contexts in the MRI scanner by placing optic fibres and air-delivery tubing over the MRI stereotaxic frame. MRI-compatible optical fibres (1.5 mm diameter, Chinly, China) were arranged to constitute a single or four linearly arranged light sources, thus reproducing the visual patterns of the CPP box. The optical fibres’ ends were placed within the focal distance of the rat’s eye, and the olfactory cues were delivered near the rat’s nose via air tubing connected to an olfactometer (Fig. 2A, left). Visual and odour stimuli were synchronized with MRI scanner trigger-out, automatically presented as pairs, synchronized using an Arduino board, thus mimicking the contextual arrangements present in the behavioural apparatus (Fig. 2A, right). We will refer to this stimulus presentation as the cue-based fMRI sequence.

Baseline structural and resting-state fMRI sequences were acquired for a few days before and a few hours following the post-conditioning session (matching the time of day when they would normally receive morphine throughout the conditioning sessions), ∼2 weeks within each other (according to the timeline depicted in Fig. 2A). Each animal was placed in the MRI frame on only two occasions. PP was assessed before the cue-based fMRI sequence to ensure accurate measurement of behaviour. Since the interval between the two procedures was ∼4 h, extinction processes are likely to play a negligible role in the fMRI measurements.^38^

Brain fMRI was carried out using a high-resolution 9.4 T small bore animal scanner (BioSpec 94/20, Bruker, MA, USA). A rat head–adapted room temperature surface coil was combined with a volume transmission coil, and acquisitions were made using Paravision 6.0.1 software (PV6, Bruker). Anatomical images were acquired using turbo rapid acquisition with relaxation enhancement (Turbo-RARE) T_2_-weighted sequence (22 continuous slices with 0.9 mm, TE = 33.00 ms, TR 2.5 sec, resolution = 0.098*0.098 mm and averages = 2), as a standard procedure in rodent structural imaging, allowing us to keep an adequate acquisition time at high field. Using free induction decay echo-planar imaging (FID–EPI) and a repetition time (TR) of 2 s, fMRI data were acquired in two blocks for the post-behaviour imaging session, 30 min after medetomidine and isoflurane clearance: resting-state fMRI for 10 min, with a 2 min interval before initiating the cue-based fMRI. To monitor brain activity during contextual memory recall, we targeted the low-frequency dynamics of BOLD signal for an extended temporal window, thus dividing visual–olfactory cue presentation into two continuous 10 min blocks, to mimic a time frame of free exploration, memory reactivation and conditioned response, to which animals were exposed in the CPP apparatus on the post-conditioning (or test) day. For all animals, the first 10 min block presented corresponded to the ‘cinnamon + white light 4-LED array’, and the second to the ‘thyme + white light point’ contexts. Both contexts were separated by a 5 min inter-block pause. Timelines for the fMRI experiments can be found in Fig. 2B. Although the two contexts were presented in the same order, the associated outcome (context valence) counterbalanced the context presentation order. Consequently, six rats first encountered the morphine-paired cues, and the other six were first exposed to the saline-paired cues. Sixteen axial slices of 0.9 mm thickness were recorded to cover the main areas of interest in the brain (echo time/repetition time = 15/2000 ms, spatial resolution = 0.234 * 0.234 * 0.9, matrix 128 * 128, field of view (FOV) 30 * 30 mm, bandwidth = 400 000 Hz). After imaging procedures, animals received a subcutaneous bolus injection of 0.1 mg/kg atipamezole (Antisedan, Pfizer, Karlsruhe, Germany), to counteract the effects of medetomidine, and were placed in a recovery box for post-scan monitoring until they were fully awake.

### fMRI data pre-processing and analysis

Imaging data were pre-processed using SPM12 (statistical parametrical mapping) toolbox for MATLAB (R2019b). Pre-processing steps included realignment of images to the first volume (or scan) of each acquired sequence, normalization to a common rat brain template (affine transformation) to account for individual anatomical and positional differences and smoothing for signal-to-noise ratio improvement (Gaussian kernel with FWHM of twice voxel size). A final filtering step using a 0.01–0.1 Hz band-pass filter was performed using the RESTplus toolbox (v1.25_20210630) for MATLAB to extract low-frequency fluctuations specific to the BOLD signal.

For functional connectivity (FC) analysis, we selected some regions for seed-based and region of interest (ROI)–based static connectivity analysis. Seed regions were selected from the cortico-limbic system involved in memory processing (encoding and retrieval), affective state and valence/salience assignment, including three amygdala nuclei [anterior cortical amygdaloid nucleus (ACo), basomedial (BMA) and central (CeA) amygdala], the intermediate portion of the lateral septum (LSI), lateral habenula (LHb), VTA, two HIPP sub-regions (DG and CA1 subfield) and two cortical structures, cingulate (Cg) and retrosplenial cortices(RSCs). ROIs were manually defined by drawing masks of varying voxel size over these regions bilaterally across the template brain images using MRIcron (RRID:SCR_002403) software. Depending on the region's antero-posterior extension, some masks spanned across multiple slices and were combined into a single mask. Adobe Photoshop (CC 2019) was used to confirm correct mask positioning by superimposing Paxinos and Watson's rat brain atlas figures^37^ over the sixteen template functional brain slices covering the whole brain.

For ROI-based analysis, a MATLAB-written script was developed to extract time series data from individual ROIs as well as pairwise Pearson's correlation coefficients from all possible pairs of ROIs. FC matrices were *z*-transformed (Fisher's *z*-score) and obtained for baseline (pre-CPP) and post-CPP resting-state sequences. A separate set of matrices was extracted from the ‘context recalling’ sequence and represented as correlation matrices of functional connectivity for conditions ‘morphine’ and ‘saline’. To ensure that animals perceived contextual cue presentation under (light) sedation, we also performed pairwise comparisons between resting-state and cue-state sequences (pre-CPP and post-CPP resting-state individually compared to morphine- and saline-paired contexts). FC matrices were also extracted for other limbic and sensory information-processing brain regions (Supplementary Fig. 4).

For seed-based analysis, the same user-defined ROI masks were used as seeds for voxel-wise whole-brain connectivity analysis. The RESTplus toolbox retrieved zFC maps for each subject which were later combined into group mean maps for each of the four study conditions. Positive voxel *t* values indicated first condition greater than the second; for resting-state analysis, post-CPP corresponds to condition 1 and pre-CPP to condition 2. For cue-based analysis, ‘morphine context’ represents condition 1 and ‘saline context’ condition 2. In the end, statistical maps were overlaid onto template anatomical T_1_ brain images. An in-house MATLAB script was used to extract mean functional connectivity data from each seed to measure their intra-network relevance across the four study conditions.

Graph theoretical representations of the cortico-limbic network under study were extracted from the MATLAB-based visualization toolbox BrainNet Viewer (v.1.7, Xia *et al*., 2013). Node files containing ROI mask *x–y–z*-coordinates, node centrality degree and its corresponding colour coding were created. For graph representation, we extracted only unilateral coordinates of our ROI masks from MRIcron. Node centrality degree was calculated as the sum of all connections each node (ROI mask) had in our undirected network, based on the work by Centeno el al.^39^ Node colour was defined from a direct conversion of nodal degree into the jet colour map code in MATLAB (scale limits were set based on the least and most central nodes in our analysis). Edge files contained the correlation matrices extracted from our ROI-based analysis. Purely for visualization purposes, edge threshold was arbitrarily set at a *z*-score of 0.2. For the surface file, we used the SIGMA Rat Functional Imaging EPI Brain Template.^40^

To assess whether baseline brain functional connectivity predicts performance in the CPP test, we correlated the CPP-deltas of each individual animal against the ROI-based correlation coefficients (or FC) of all possible ROI pairs for the resting-state conditions and context recalling conditions, under the hypothesis that (i) brain connectivity at pre-CPP could predict performance in the CPP test and (ii) performance in the CPP test could be correlated to connectivity strength during contextual cue exposure. Our general prediction for the latter was that subjects scoring higher in the CPP test would have a higher functional connectivity in memory-related and/or valence-assignment regions during presentation of morphine-paired contexts compared to the level of connectivity during exposure to saline-paired contexts. For rats that scored low or negative deltas, either the reverse or the absence of a correlational effect would be observed.

The activity analysis code in Python and the connectivity analysis MATLAB scripts are available on GitHub (https://github.com/joana-g-ribeiro/rat-addiction-fMRI-).

### Statistical analysis

Activity analysis was performed in Python using Nipy, and connectivity analysis was performed in MATLAB. For activity analysis, we fit a single generalized linear model (GLM), implemented in Python using Nipy,^41^ to our continuous BOLD data by concatenating the recordings of all 12 rats accounting for the context presentation order. The two 10 min blocks were partitioned into 30 s windows (total = 40 windows) to be sensitive to changes in response over the long duration of contextual cue presentation. A regressor was added to the design matrix for each window using a rat-adjusted haemodynamic response function.^42^ The six realignment parameters and the whole-brain mean signal of individual rats were also added as nuisance regressors. We considered contrasts for individual windows and conditions (contexts) in addition to differences between conditions for matched windows (e.g. first-window saline minus first-window morphine). All comparisons were FDR-corrected with a significance level set at 0.05 and a cluster size threshold of 16 voxels (0.79 mm^3^).

For connectivity analysis, Pearson's correlation values were extracted using MATLAB-written scripts and compared at the ROI pair level between two conditions (resting-state, pre-CPP versus post-CPP; context recalling, saline context versus morphine context) using paired *t*-tests with FDR corrections. *P*-value matrices (uncorrected and FDR-corrected) displaying statistically significant differences between the two acquisition moments were also computed. We performed seed-based analysis using the RESTplus toolbox and retrieved *z*-scored FC maps for each subject, which was later combined into group mean maps for each of the four study conditions. Pairwise paired *t*-tests were performed on these images, and t maps were obtained and corrected for multiple comparisons using the Gaussian Random Field Theory procedure (one-tailed with voxel- and cluster-level *P-*values set at 0.05 were used as statistical map correction parameters). For correlation analysis between FC and CPP-delta, we performed a simple linear regression and plotted the CPP-delta against the ROI-based correlation coefficients of all possible ROI pairs for the four study conditions as well. Pearson's R were computed along with significance values.

## Results

### Validation of a new MRI-compatible conditioned place preference protocol

Animals displayed a preference for the morphine-paired compartment during post-conditioning [Fig. 1C, two-way repeated measures ANOVA, compartment effect, *F*_(2,33)_ = 2.77, *P* = 0.0003; Bonferroni-corrected multiple comparisons and mean time (s) ± SEM, post-conditioning saline (264.58 ± 18.15) × post-conditioning morphine (376.08 ± 29.24): *P* = 0.0047]. Throughout conditioning, morphine administration did not impact locomotor activity as measured by the cumulative walking distance in the enclosed compartments [two-way repeated measures ANOVA, no session × compartment interaction: *F*_(5, 110)_ = 0.77, *P* = 0.574], but a strong effect was found on the mean speed (without considering rest time) as it gradually increased and significantly peaked on the last conditioning day [two-way repeated measures ANOVA, session × compartment: *F*_(5, 110)_ = 12.93, *P* < 0.0001; Bonferroni-corrected multiple comparisons and mean (cm/s) ± SEM: day 6 saline-paired compartment (4.712 ± 0.114) × day 6 morphine-paired compartment (7.224 ± 0.437); *P* = 0.0006); day 5 morphine-paired compartment (5.853 ± 0.437) × day 6 morphine-paired compartment (7.224 ± 0.437); *P* < 0.0001]. Those rats showing both an increased time spent in the morphine-paired compartment from pre- to post-conditioning and a >50% permanence therein were classified as ‘learners’ (7/12; Fig. 1E, blue dots). Five of the 12 rats showed a 7–27% decrease in time spent in the morphine-paired compartment in the test phase and were considered ‘non-learners’ as a result (Fig. 1E, orange dots).

By computing the difference between the time spent in the morphine-paired compartment post- versus pre-conditioning (in s), we can attribute to each individual animal a CPP-delta value reflecting how effective was the association between context and morphine. We did not find a correlation between CPP-delta and the two locomotion parameters evaluated in the present study (Fig. 1F top; distance travelled: *R*^2^ = 0.094; *F*_(1,10)_ = 1.041, *P* = 0.332; Fig. 1F bottom; mean speed: *R*^2^ = 0.003; *F*_(1,10)_ = 0.028, *P* = 0.871), indicating that our morphine-induced PP did not result from the locomotor reactivity to morphine.

Despite the CPP score variability, we performed a group-level analysis to identify overall patterns of activity and connectivity linked to each type of context and later assessed how the magnitude of CPP was related to individual brain connectivity.

### Morphine- and saline-associated contextual stimuli reactivate common, and specific, hippocampal–prefrontal–mesolimbic areas around stimulus onset

MRI activity maps revealed both context-specific and context-general activity clusters across the whole brain in the first 30 s of cue presentation (Fig. 2C). Morphine-associated stimuli presentation (Fig. 2Ci) resulted in increased activity in prefrontal, HIPP and para-HIPP regions, entorhinal cortex and connected subcortical areas (see Table 1 for full details). These maps were generated using a single GLM, with an intercept which captures the average signal over the whole time series, and a few regressors to allow adjustment for the individual rat haemodynamic response and to align temporal and spatial signals for each voxel. The mean signal of individual rat brains was also added to the design matrix. If together the regressors contribute to a change beyond the average time series signal, they will be significantly non-zero and appear in the maps as colours in the red and blue scales, representing positive and negative changes, respectively. Saline-associated stimuli increased activity over the antero-posterior motor cortex, the somatosensory cortex, medial septum, RSC, periaqueductal grey, ventral HIPP (vHIPP) and inferior colliculus (Fig. 2Cii; Table 1). Activation of some of these regions has been shown in response to withdrawal-related cues in the rat, namely, the thalamic and hypothalamic regions,^43^ but at least the activation of cognitive and memory regions seems to be specific to morphine memory retrieval, rather than responses to withdrawal states.

**Table 1:** Mean BOLD signal changes in response to saline and morphine-paired cues in the first 30 s of stimuli presentation in the whole brain

Brain regions	BOLD signal change	Morphine cue-specific	Saline cue-specific	Common to morphine and saline cues
Olfactory nuclei	↑			Anterior olfactory nuclei (lateral and ventral subdivisions)
	↓		Nucleus of the olfactory tract (LOT)	Olfactory tubercule
Prefrontal cortex	↑	Pre-limbic (PrL), Infralimbic (IL), and Orbitofrontal (OFC) cortices		Cingulate cortex (> morphine);
	↓			
Basal forebrain	↑	Bed nucleus of the stria terminalis (BNST), Nucleus of the vertical limb (diagonal band of Broca)		Nucleus accumbens (core and shell) (> morphine), Lateral septum (LS) (> morphine), Medial preoptic area (> morphine), Dorsal striatum (posterior)
	↓	Central amygdala (CeA)	Anterior cortical amygdaloid nucleus (ACo), Basolateral amygdala (BLA)	Dorsal striatum (anterior)
Hypothalamus	↑	Anterior and posterior hypothalamic areas		
	↓			
Cerebral cortex	↑	Anterior insular, Entorhinal (medial and dorsolateral), and perirhinal cortices	Primary and secondary motor, primary somatosensory, and retrosplenial (dysgranular) cortices	Anterior cingulate (> morphine), Primary and Secondary auditory (> morphine), Secondary somatosensory, Primary and Secondary visual (> saline), Parietal association, Temporal associative, and retrosplenial cortices
	↓	Somatosensory and motor cortices	Piriform, Entorhinal (dorsolateral), and perirhinal cortices, ventral clastrum	Parietal association, posterior insular, and retrosplenial cortices
Hippocampus	↑	CA2, CA3, Dorsal subiculum	Intermediate CA1	
	↓		Ventral subiculum, parasubiculum	Posterior distal dorsal CA1, ventral CA1 (> saline)
Thalamus	↑	Medial geniculate nuclei		Nucleus reuniens, Post-thalamic nuclear group
	↓	Lateral habenula		
Midbrain	↑	Parvicellular regions of the red nucleus, ventral tegmental area (parabraquial pigmented nucleus), Anterior pretectal nucleus	Periaqueductal grey (dorsomedial subdivision)	Rostral ventral tegmental area (opposite hemispheres)
	↓		Periaqueductal grey (ventrolateral subdivision)	Superior and inferior colliculus, Cuneiform nucleus
Pons	↑	Pontine reticular nucleus		
	↓		Laterodorsal tegmental nucleus	Dorsal raphe, Pedunculopontine tegmental nucleus

Up and down arrows indicate positive and negative BOLD signal changes, respectively. Anatomical identification of activated clusters was achieved by superimposing Paxinos and Watson (2007)^37^ brain atlas figures onto the activity maps of Fig. 2Ci and ii.

Context-general activated regions tended to elicit stronger activity in response to morphine-associated stimuli. These included the cingulate cortex, the nucleus accumbens and auditory cortices (Table 1). Most of the context-general activation is bilateral. Exceptions, unilateral and in opposite hemispheres are the ventral tegmental area (morphine-associated cues, left hemisphere; saline-associated cues, right hemisphere, Fig. 2Ci and ii). We also found negative clusters throughout the whole brain, and mostly located over the insular cortex, somatosensory cortex, amygdala sub-regions, vHIPP and superior colliculus in both morphine- and saline-associated cues.

We then compared activity, still in the first 30 s window, by looking at the signal magnitude differences (Figs 2Ciii and iv; Table 2). Significant clusters of increased activity in morphine relative to saline contexts were found over prefrontal cortex, anterior cingulate and RSC, nucleus accumbens (core), hippocampus and reticular formation. Conversely, we found clusters with higher activity in saline relative to morphine contexts over the motor cortex, somatosensory cortex, caudate putamen, cingulate and RSC, hypothalamus, hippocampus, medial pretectal nucleus, visual cortex and superior and inferior colliculi. Altogether these results demonstrate that saline-associated cues activate regions involved in somatosensory processing, while morphine-associated cues activate higher cognition, limbic and memory regions.

**Table 2:** Differences in mean BOLD signal between saline and morphine-paired cue responses in the first 30 s of stimuli presentation in the whole brain

Brain regions	Difference morphine–saline cues	Difference saline–morphine cues
Prefrontal cortex	Pre-limbic (PrL), Infralimbic (IL), orbitofrontal (OFC), and Cingulate cortices	cingulate cortex
Basal forebrain	Nucleus accumbens (core subdivision)	Dorsal striatum (anterior)
Hypothalamus		Lateral hypothalamus
Cerebral cortex	Entorhinal and retrosplenial cortices	Anterior insular (dysgranular), Primary and secondary motor, Primary somatosensory, Primary and secondary visual, Parietal association, and Retrosplenial cortices
Hippocampus	Dorsal subiculum, CA1, DG, presubiculum, parasubiculum	Posterior distal dorsal CA1
Thalamus	Post-thalamic nuclear group	
Midbrain	Anterior pretectal nucleus, Reticular formation, Periaqueductal grey (ventrolateral subdivision)	Superior colliculus

Activated clusters in ‘saline cue’ condition were subtracted from ‘morphine cue’ condition, and *vice versa*, to obtain magnitude difference maps. Anatomical identification of activated clusters was achieved by superimposing Paxinos and Watson (2007)^37^ brain atlas figures onto the activity maps of Fig. 2Ciii and iv.

Significantly increased and decreased activity clusters appear primarily in the first 30 s window, and only again in the last two acquisition windows (Fig. 2D; Supplementary Fig. 2 and Tables 1 and 2). That was the case for several subdivisions of the insular cortex (granular, agranular and dysgranular), amygdala (central, basolateral and basomedial) and amygdaloid nuclei (anterior cortical, anterior area, lateral and medial amygdaloid), ventral pallidus, ventral claustrum, ventral subiculum, dorsal raphe, lateral parabrachial nucleus, superior colliculus, pre-mammillary nucleus, substantia nigra and ventral DG. Specific to saline contexts (Fig. 2Dii) were VO and LO, DS, medial pretectal nucleus, AcbC, granular and dysgranular insular cortex, substantia nigra, ventral CA1, ventral DG and MEnt, which had not appeared active in the first window. Specific to morphine contexts (Fig. 2Di), were ACo, LPtA, MPtA, ventral pallidus, ventral claustrum, pyramidal cell layer of anterior CA1, dorsal raphe, LPAG and lateral parabrachial nucleus.

### Drug–context pairings promoted low-level rearrangements of resting-state cortico-limbic network connectivity

**Figure 3:**
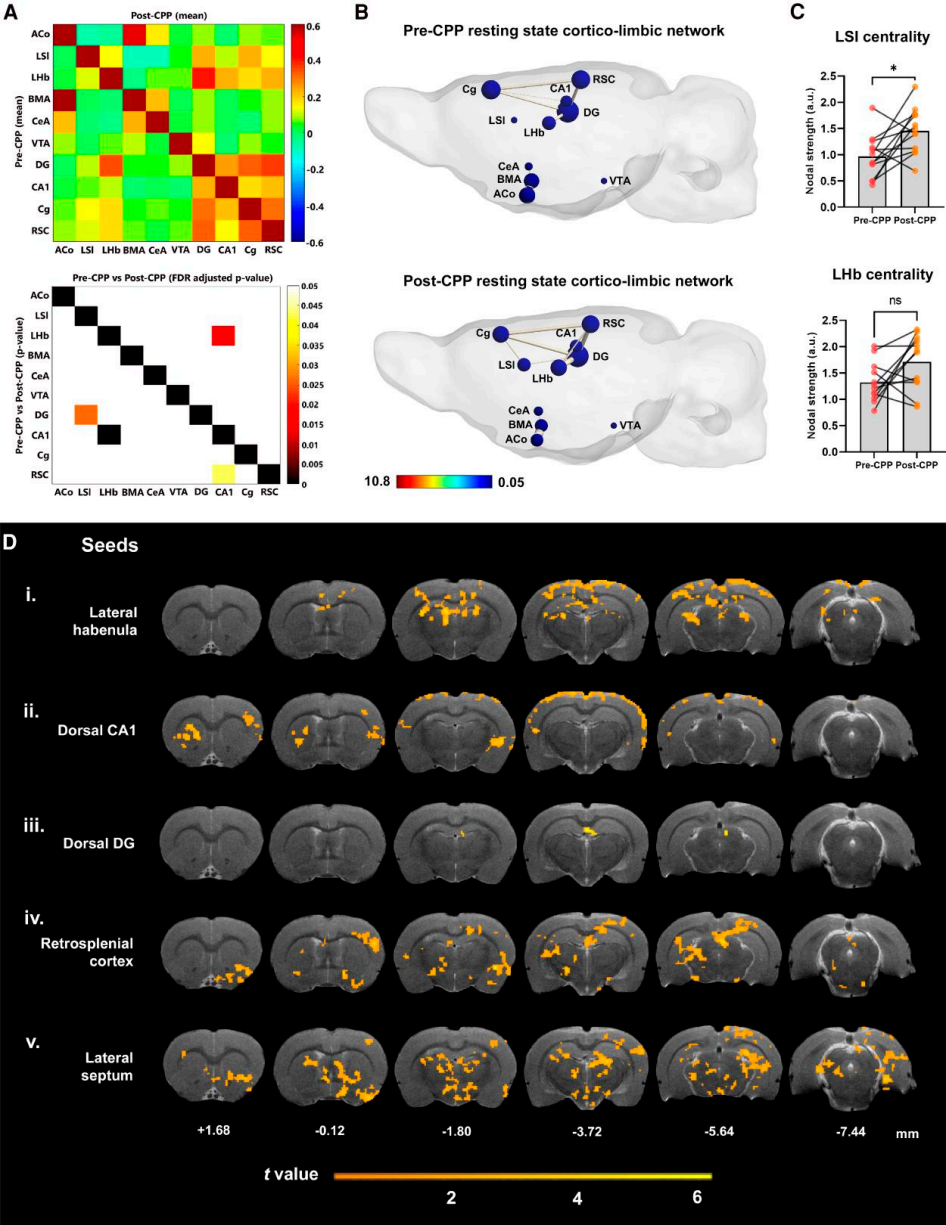
**Drug–context pairings promoted low-level rearrangements of resting-state cortico-limbic network connectivity.** (**A**) Functional connectivity (FC) increases after morphine conditioning. Upper panel: Mean FC matrices of pre-CPP (lower half) and post-CPP (upper half). X- and Y-axes represent brain regions. Colour scale represents Pearson correlations, i.e. strength of FC between each pair of brain regions. Lower panel: *P*-value matrix (lower half) representing statistically significant differences between pre-CPP versus post-CPP resting-state connectivity. FDR-adjusted *P*-value representing statistically significant differences between pre-CPP versus post-CPP resting-state connectivity (upper half). Colour scale represents *P* values (0–0.05). X- and Y-axes represent brain regions. Abbreviations: ACo, anterior cortical amygdaloid nucleus; LSI, lateral septum intermediate; LHb, lateral habenula; BMA, basomedial amygdala; CeA, central amygdala; VTA, ventral tegmental area; DG, dentate gyrus; CA1, CA1 region of the hippocampus; Cg, cingulate cortex; RSC, retrosplenial cortex. *n* = 12 rats. (**B**) Graph theory representation of the putative cortico-limbic network in pre- and post-conditioning at rest. Colour scale represents node colour, which was defined from a direct conversion of nodal degree into the jet colour map code in MATLAB (scale limits were set based on the least and most central nodes in our analysis). *n* = 12 rats. (**C**) Plots depict centrality levels of two nodes of the network, the LSI and the LHb. Values are presented as mean nodal strength ± SEM. *P < 0.05 (Paired t test). *n* = 12 rats. (**D**) Representative anatomical scans (T_2_-weighted anatomical MRI image of our template brain) display mean statistical t maps of resting-state connectivity for five regions within our putative cortico-limbic network, converted into seeds for seed-based analysis (pairwise paired *t*-test, with Gaussian random field theory correction procedure for multiple comparisons; one-tailed and voxel- and cluster-level *P*-values set at 0.05). Colour scale represents *t*-value, i.e. strength of functional connectivity of the seed region with all voxels in the brain. Coordinates under each selected brain section represent Paxinos and Watson's^37^ rat brain atlas coordinates (in mm). *n* = 12 rats.

We hypothesized that morphine–context pairings during CPP would impact neural processing of sensory, mnemonic and emotional information, revealed by overall changes in correlated BOLD signal across pertinent neural populations. We thus focused on pairwise comparisons of resting-state BOLD signal amongst 10 empirically selected regions of a putative cortico-limbic network, before and after behavioural manipulations. In both pre- and post-CPP, our ROI-based analysis revealed significantly correlated activity across CA1, DG, Cg and RSC, regions involved in spatial contextual memory processing and consolidation (Fig. 3A top, *z*-scored matrix). This is consistent with these regions’ well-known engagement in the default mode network (DMN), which is involved in episodic memory.^44^ In addition, we found that the two amygdala nuclei were correlated with each other and with one of their cortical counterparts, ACo, in both conditions. When we compared the set of correlation coefficients for each ROI pair between pre- and post-CPP, we found a significant difference between the pre- versus post-connectivity in the LHb–HIPP CA1 pair (Fig. 3A bottom, paired *t*-test, pre- × post-CPP Pearson's correlation coefficients, FDR-corrected *P* = 0.014).

We then looked at network-level interactions by using graph theoretical analysis, and analysing nodes and edges as proxies for brain region activity levels and functional connections between region pairs, respectively.^45^ Nodal degree, also known as centrality, reflects the connection density of each individual brain region, e.g. its importance in a given functional network.^39^ Here, we measured centrality as the sum of all correlation coefficients of an individual region with every other region in the proposed network and found that the LSI exhibits a significant increase in centrality from pre- to post-CPP (Fig. 3B bottom and C top, two-tailed paired *t*-test, *t* = 2.735, df = 11, *P* = 0.019), which was not the case for LHb (Fig. 3C bottom, two-tailed paired *t*-test, t = 2.069, df = 11, *P* = 0.063). This suggests that both positive and negative affect processes, engaging episodic memory and reward (or withdrawal)-related regions, may occur throughout the morphine-conditioning protocol, in alignment with the acute shifts between rewarding and aversive states during the development of drug addiction.

To expand our analysis beyond pairwise correlations in cortico-limbic circuits, we selected the five regions whose connectivity changed from pre- to post-CPP and used them to seed voxel-wise whole-brain connectivity analysis: LSI, LHb, CA1, DG and RSC. Statistical maps in Fig. 3 show, for each seed region, Gaussian-corrected one-tailed *t*-maps for post-CPP versus pre-CPP comparisons (with a voxel and cluster threshold set at 0.05), highlighting the regions whose activity exhibited higher correlation in the post-CPP resting-state versus pre-CPP (Fig. 3D).

In this analysis, the LHb, a region involved in aversive learning, is highly correlated with medial mesocortical cingulate and retrosplenial areas, and with visual, somatosensory, medial and lateral parietal regions, and dorsal CA1, in line with the increase in resting-state connectivity observed in the ROI-based analysis explained above (Fig. 3Di). Thus, following CPP, there seems to be a dissociated emergence of two, known, distinct networks, an affective one centred in the LHb, and a spatial–contextual–mnemonic one centred around HIPP–RSC.

Notably, the LSI, the strongest non-HIPP target of HIPP CA3 outputs, and a significant source of inputs to VTA, exhibited increased post-CPP correlations with the highest number of brain areas, consistent with the increases in centrality observed in the ROI-based analysis explained above in post-CPP resting state (Fig. 3Dv). Here, it is noteworthy the connectivity with regions of the multi-sensory integration and spatial contextual processing network, and with affective and sensory processing-related regions such as nucleus accumbens core, hypothalamic and thalamic nuclei, BNST, LHb, PAG and VTA. In this case, LSI seems to emerge as the central hub of a functional network linking the two dissociated emergent networks, affective and spatial–contextual–mnemonic, discussed above.

Other seed region connectivity can be found in Supplementary Fig. 3.

### Valences of context share a common circuit but rely on affective state for discrimination

Why are there two functionally distinct networks emerging independently upon morphine-induced CPP and linked by LSI?

The olfactory and visual systems, responsible for processing and integrating sensory information, were expected to be highly engaged during presentation of the two contextual cue modalities. We investigated their intra-system correlation coefficients in the four study conditions (pre- and post-CPP resting state, and saline- and morphine-associated cues presentation) and found a significant difference between the pre- versus post-CPP connectivity in the anterior olfactory nucleus (AON)–nucleus of the lateral olfactory tract (LOT) pair (Supplementary Fig. 4, paired *t*-test, pre- × post-CPP Pearson's correlation coefficients, FDR-corrected *P* = 0.047). No further differences were found between pre- versus post-CPP and saline- versus morphine-associated cue connectivity in olfactory and visual regions.

We then compared their coactivity with cognitive/emotional regions by randomly selecting four cortico-limbic network regions (see above) and two entorhinal cortex regions, which are well-known gateways for sensory information entering in the hippocampus.^46,47^ Here, we found that presentation of morphine-associated cues elicited a significant change in the patterns of coactivity when compared to post-CPP resting-state connectivity. Most strikingly, connectivity within visual regions and their connectivity with CA1 and DG were both significantly altered (Supplementary Fig. 4Fi, morphine versus post-CPP matrix), which was not the case for olfactory regions (Supplementary Fig. 4Ci, morphine versus post-CPP matrix), suggesting that visual contextual sensory encoding dominates the initial acquisition of a contextual representation, and likewise its subsequent association with affective stimuli.

**Figure 4:**
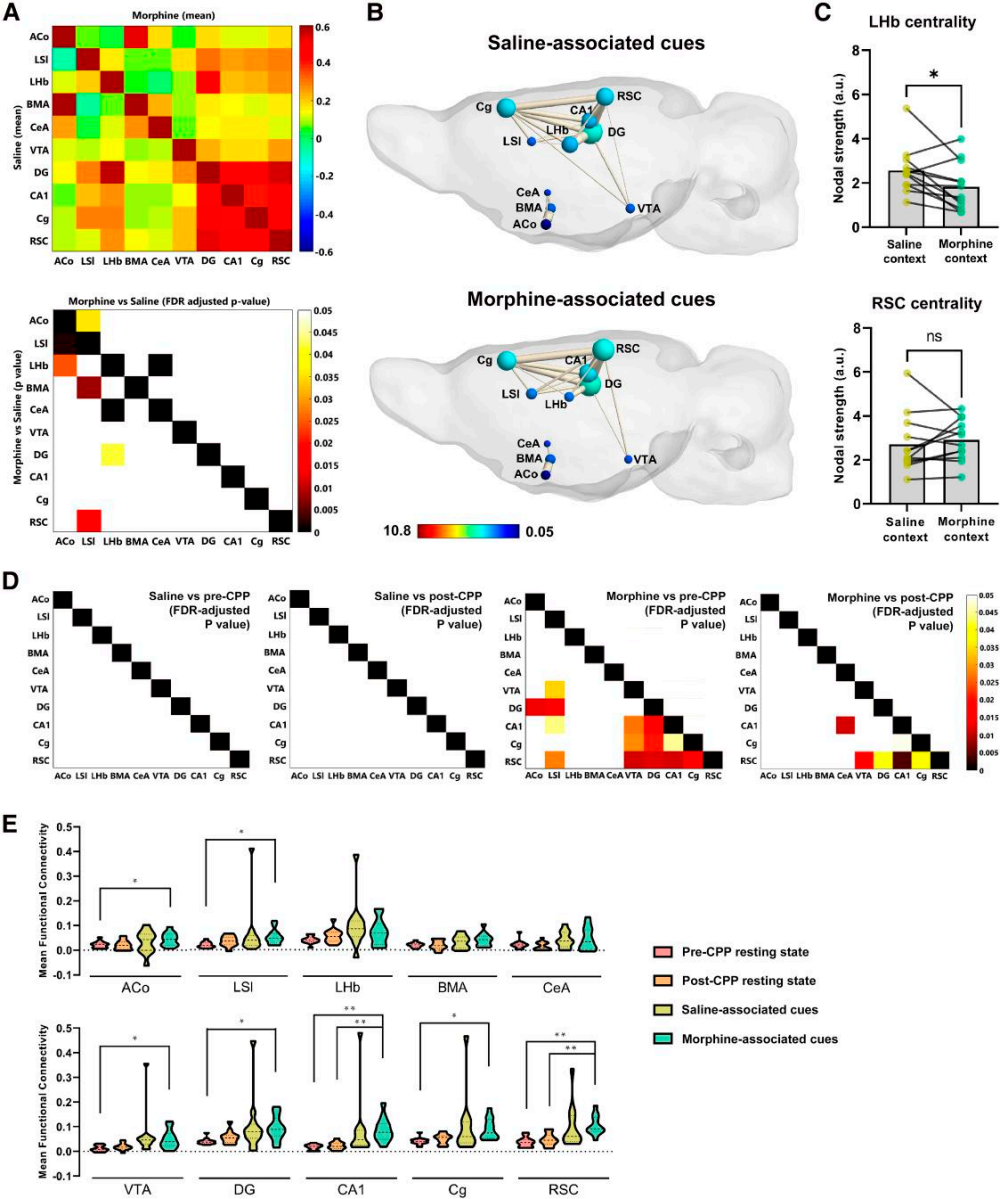
**The same limbic circuits are involved in the retrieval of distinctly valenced contexts, but discrimination likely depends on affective state.** (**A**) FC between saline- and morphine-paired contexts. Upper panel: Mean FC matrices of saline (lower half) and morphine (upper half) contexts. X- and Y-axes represent brain regions. Colour scale represents Pearson correlations, i.e. strength of FC between each pair of brain regions. Lower panel: *P*-value matrix (lower half) representing statistically significant connectivity differences between saline versus morphine contexts. FDR-adjusted *P*-value representing statistically significant connectivity differences between saline versus morphine (upper half). Colour scale represents *P*-values (0–0.05). X- and Y-axes represent brain regions. Abbreviations: ACo, anterior cortical amygdaloid nucleus; LSI, lateral septum intermediate; LHb, lateral habenula; BMA, basomedial amygdala; CeA, central amygdala; VTA, ventral tegmental area; DG, dentate gyrus; CA1, CA1 region of the hippocampus; Cg, cingulate cortex; RSC, retrosplenial cortex. *n* = 12 rats. (**B**) Graph theory representation of the putative cortico-limbic network during exposure to saline and morphine contexts. Colour scale represents node colour, which was defined from a direct conversion of nodal degree into the jet colour map code in MATLAB (scale limits were set based on the least and most central nodes in our analysis). *n* = 12 rats. (**C**) Plots depict centrality levels of two nodes of the network, the LHb and the RSC. Values are presented as mean nodal strength ± SEM. *P < 0.05 (Paired *t*-test). *n* = 12 rats. (**D**) FDR-adjusted *P*-value matrices representing statistically significant connectivity differences saline context exposure and the two resting-state conditions (two left matrices), and morphine context exposure and the two resting-state conditions (two right matrices). Colour scale represents *P*-values (paired *t*-test, 0–0.05). *n* = 12 rats. (**E**) Mean FC plots representing mean FC of the 10 seed regions with other voxels in the brain for all fMRI conditions. *P < 0.05, **P < 0.01 (repeated measures one-way ANOVA). *n* = 12 rats.

Having found an activity network whose connectivity stores the representation of CPP, we then asked whether such network specifically retrieves distinct contextual representations when the animal is presented with the sensory cues that define each opposed valence context. For this, we analysed the synchronicity across such network during a 10 min presentation of either saline or morphine-associated cues. In agreement with the data shown above, we observed strong correlations between spatial contextual and episodic memory processing regions (Fig. 4A top, *z*-score matrix, DG, CA1, Cg and RSC), and between these and the LSI, LHb and VTA, with no significant differences between the two contexts (Fig. 4A bottom, *P*-value matrix).

Neural activity in two amygdala regions was negatively correlated with that in either LSI or LHb when animals were presented with sensory cues defining opposed valence contexts. Namely, presentation of saline contextual cues resulted in a negative correlation between the cortical amygdaloid nucleus and the intermediate LSI, whereas in the morphine case, a negative correlation was found between the central nucleus of the amygdala and the LHb (Fig. 4A bottom, paired *t*-test, FDR-corrected z = −0.057, *P* = 0.036; *z* = −0.013, *P* = 0.00068, respectively). We then analysed, as before, the average centrality of each of the candidate regions during contextual presentations. We found that exposure to morphine-associated cues globally decreased centrality in the LHb, when compared to the one obtained during saline-associated context exposure (Fig. 4B and C; two-tailed paired *t*-test, t = 2.566, df = 11, *P* = 0.026). Of all candidate regions analysed, the retrosplenial cortex (RSC), highly connected with a cortico-limbic network involved in ego and allocentric contextual encoding, exhibits the highest increases in correlations with most of this network on exposure to morphine-associated cues relative to resting-state conditions (Fig. 4D two right matrices), but no increases in centrality when compared with saline-associated cues (Fig. 4C bottom, two-tailed paired *t*-test, *t* = 0.716, df = 11, *P* = 0.489). Interestingly, network connectivity on exposure to saline-associated cues did not differ from that observed in resting-state conditions (Fig. 4D two left matrices). This reflects a possible role of the RSC in storing CPP cues of opposed valence, while discrimination may rely on inputs from or outputs to regions more dedicated to affective processing, like the LSI or the LHb.

Several other regions exhibited increased functional interconnectivity when presented with morphine-associated contexts relative to resting-state conditions, namely, the LSI, HIPP DG and CA1, Cg and VTA (Fig. 4D, two right matrices). Such enhanced recruitment of spatial–contextual memory regions accompanied by increased connectivity with LSI strongly suggests that the above-mentioned two dissociated emerging networks, affective and spatial–contextual memory, fulfil distinct functions, linked by the mutually connected LSI. Furthermore, morphine–context retrieval elicited significant increases in brain-wide connectivity for most of the 10 seed regions in our network of interest when compared to the pre-CPP resting-state condition, except for LHb, BMA and CeA. CA1 and RSC exhibited an additional difference with the post-CPP resting-state condition (Fig. 4E; repeated measures one-way ANOVA, followed by Bonferroni's multiple comparisons corrections, *P*-value threshold set at 0.05).

### Pre-conditioned place preference -state connectivity significantly correlates with behavioural conditioned place preference-delta

**Figure 5:**
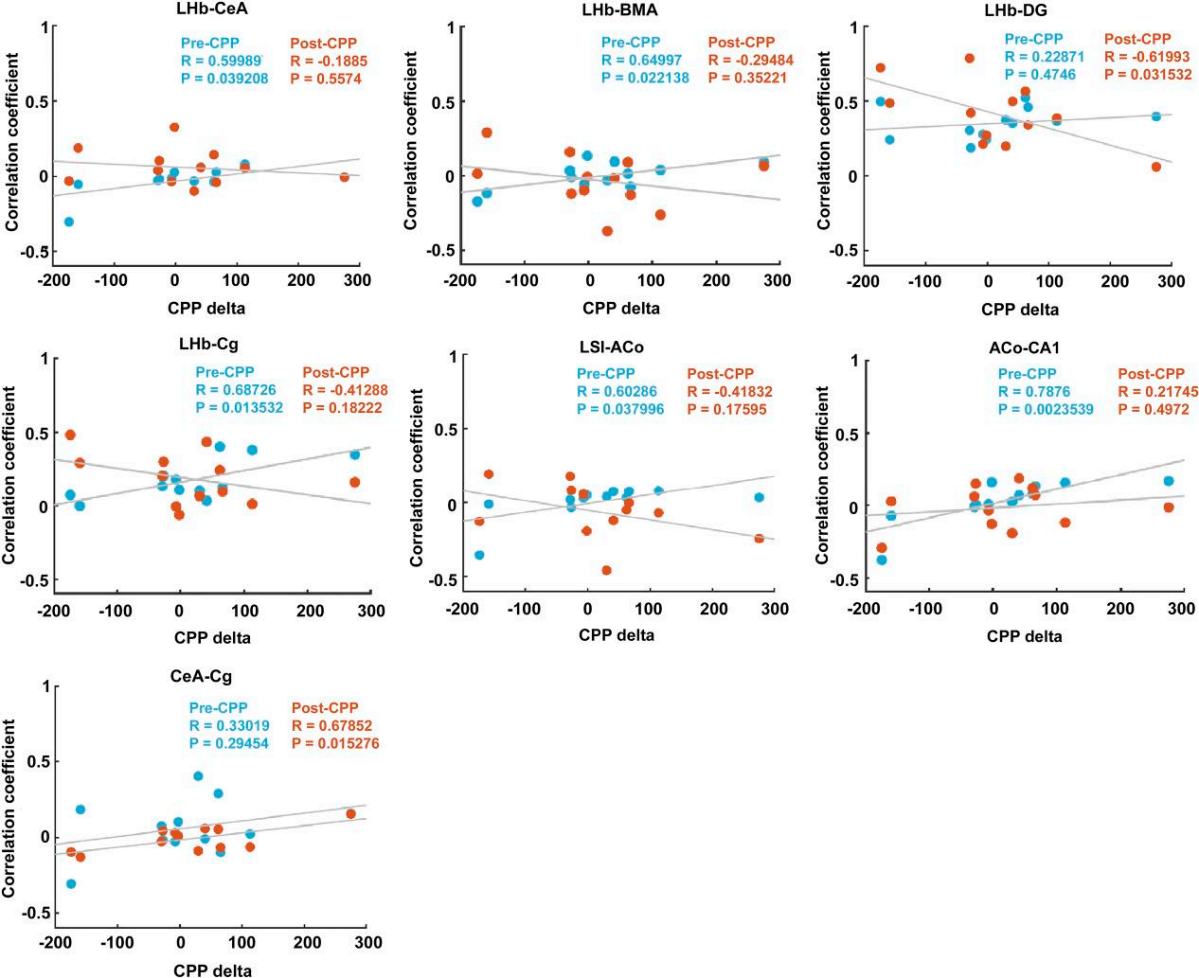
**Resting-state habenular and amygdala subcircuitry predicts scoring levels in the morphine-CPP test.** Statistically significant correlation (Pearson correlation) plots between resting-state connectivity in the indicated regions of the network and CPP-delta. Values represent the CPP-delta and the correlation coefficient for each individual animal in pre-CPP (blue) and post-CPP (orange) conditions. *R*^2^ and *P*-values are indicated in each panel. *n* = 12 rats. Abbreviations: ACo, anterior cortical amygdaloid nucleus; LSI, lateral septum intermediate; LHb, lateral habenula; BMA, basomedial amygdala; CeA, central amygdala; Cg, cingulate cortex; VTA, ventral tegmental area; DG, dentate gyrus; CA1, CA1 region of the hippocampus; Cg, cingulate cortex; RSC, retrosplenial cortex.

We found a considerable level of variability in CPP scores in our group of rats that was not directly linked to a locomotor response to morphine. We thus hypothesized that this variation in the development of CPP reflected individual intrinsic connectivity of the neural circuitry analysed above. To test this hypothesis, we performed simple linear regression on every possible pair of regions within our putative cortico-limbic network and plotted the *z*-scored connectivity for each resting-state condition against the individual CPP-deltas. Of the 45 connected pairs hypothesized, we found a significant regression in 7 pairs, including the regions found to be changed before: LHb, CeA, LSI and ACo. Four of these pairs included the LHb, a region seen before (above) to exhibit increased resting-state connectivity and decreased centrality during morphine context cue presentation and a negative correlation with the amygdala (Fig. 5).

Resting-state connectivity before CPP was found to be significantly correlated with CPP-delta, indicating that some animals are more prone to morphine conditioning. This correlation was positive in the seven region pairs, but only significant in five: LHb–CeA, LHb–BMA, LHb–Cg, LSI–ACo and ACo–CA1. This result is even stronger than the connectivity after CPP. Here, except in the case of LHb–DG and CeA–Cg pairs, all other regions showed weaker post-CPP regressions, usually accompanied by a negative regression slope and increased data dispersion. Animals with increased post-CPP connectivity usually exhibited a lower behavioural CPP-delta and *vice versa*. The fact that pre-CPP connectivity, namely, within the habenula and amygdala regions, is the best predictor of future CPP-delta, with post-CPP connectivity correlated with behavioural performance only for two pairs of regions (LHb–DG and CeA–Cg), strongly suggests that some animals are more pre-disposed to CPP by virtue of their individual neural circuit architecture and perhaps plasticity states. This raises the interesting possibility that some animals have a higher susceptibility for drug addiction, by virtue of their brains being somehow richer in its neural determinants.

**Figure 6:**
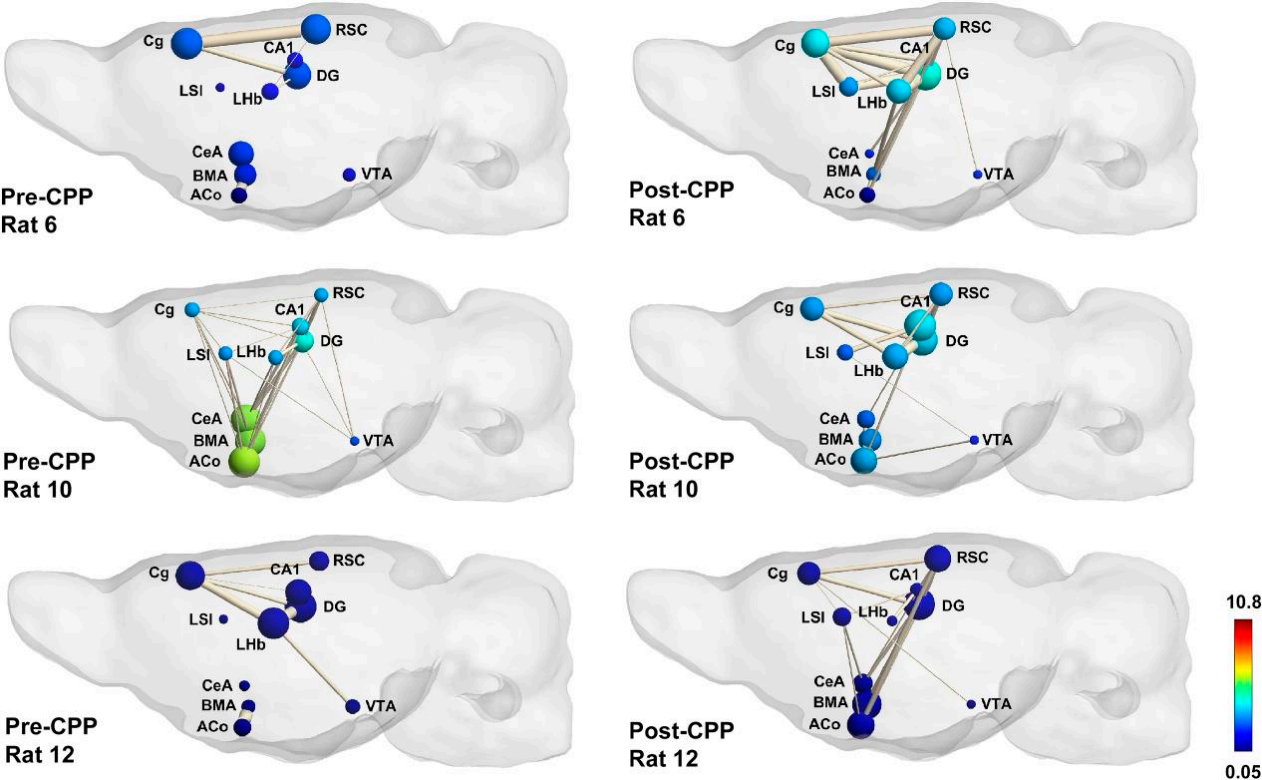
**Resting-state habenular and amygdala subcircuitry predicts scoring levels in the morphine-CPP test—putative cortico-limbic network.** Graph theory representation of the putative cortico-limbic network in pre- and post-conditioning at rest for three border animals, two bad (rats 6 and 10) and one good learner (rat 12). Colour scale represents node colour, which was defined from a direct conversion of nodal degree into the jet colour map code in MATLAB (scale limits were set based on the least and most central nodes in our analysis). Abbreviations: ACo, anterior cortical amygdaloid nucleus; LSI, ateral septum intermediate; LHb, ateral habenula; BMA, basomedial amygdala; CeA, central amygdala; Cg, cingulate cortex; VTA, ventral tegmental area; DG, dentate gyrus; CA1, CA1 region of the hippocampus; Cg, cingulate cortex; RSC, retrosplenial cortex.

The above point is further illustrated if we focus on three animals of the group (Fig. 6). We refer to them as border animals, as they scored highest (rat 12, CPP-delta = 275) and lowest (rat 6, CPP-delta = −159; rat 10, CPP-delta = −174) in the CPP test. Their individual graph theoretical representations of putative cortico-limbic network activity reveal distinct connectivity patterns. Centrality of amygdala sub-regions in pre-CPP resting state is observably higher in the two lowest scoring animals than in the highest scoring animal. The post-CPP resting-state amygdala centrality slightly changed, and we observed a higher correlation of amygdala and episodic memory regions in the latter rat. LHb centrality was also visibly different between the highest and lowest scoring animals, having, respectively, decreased and increased its degree from pre- to post-CPP. Episodic memory-related regions did not seem to show a marked difference in their centralities between animals and resting-state conditions, suggesting that CPP development, although dependent on two networks, one spatial–contextual–mnemonic and another affective, might depend on intrinsic connectivity among affective/emotional regions for its expression (acquisition and recall), and on spatial–contextual–mnemonic network for its persistence and (maybe) relapse.

### Connectivity on exposure to saline-associated cues is highly correlated with morphine– conditioned place preference test scoring

**Figure 7:**
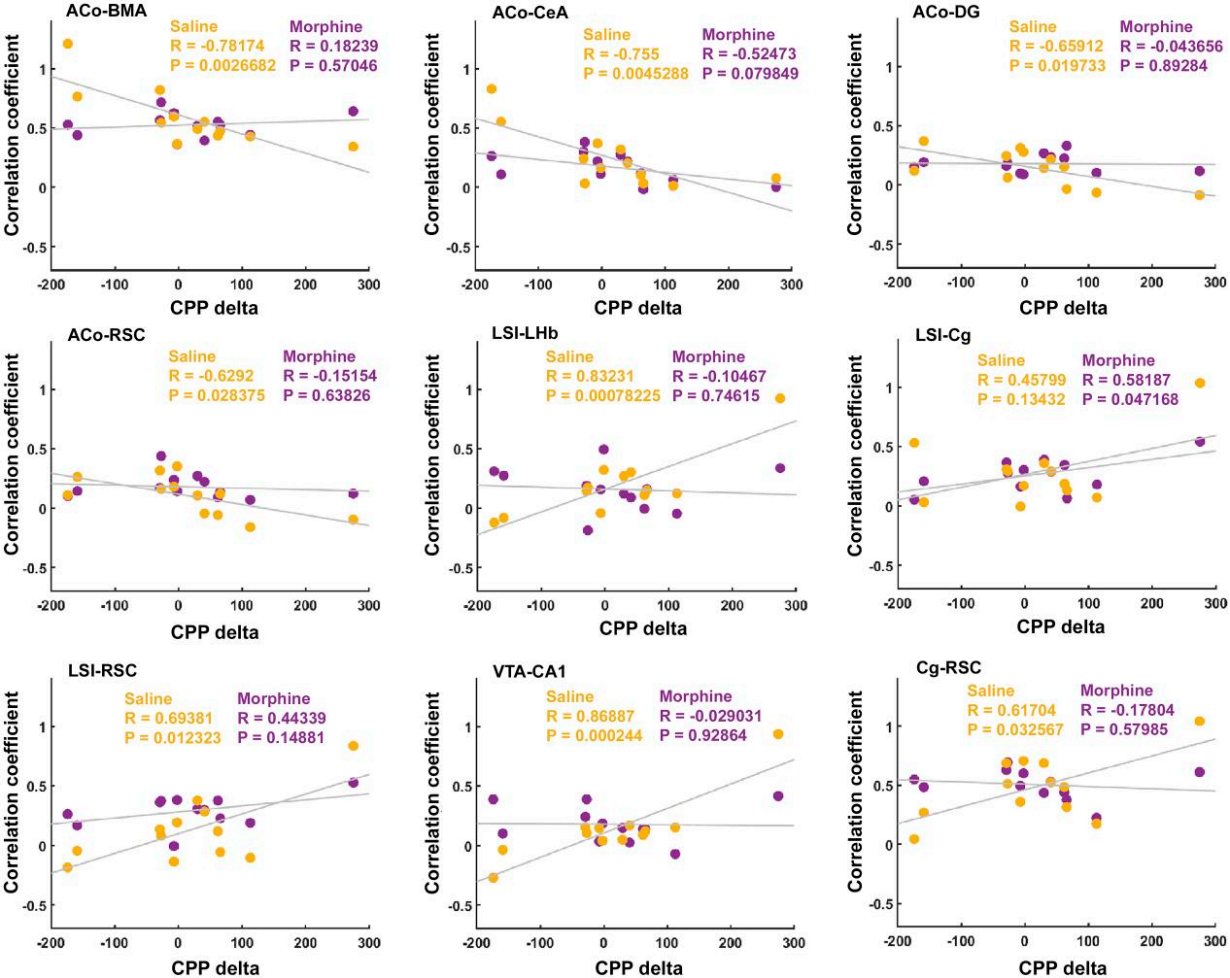
**Connectivity during saline context exposure is the better predictor of morphine-CPP test scoring.** Statistically significant correlation (Pearson correlation) plots between connectivity during exposure to saline and morphine contexts in the indicated regions of the network and CPP-delta. Values represent the CPP-delta and the correlation coefficient for each individual animal in saline (yellow) and morphine (magenta) context conditions. *R*^2^ and *P*-values are indicated in each panel. *n* = 12 rats. Abbreviations: ACo, anterior cortical amygdaloid nucleus; LSI, lateral septum intermediate; LHb, lateral habenula; BMA, basomedial amygdala; CeA, central amygdala; Cg, cingulate cortex; VTA, ventral tegmental area; DG, dentate gyrus; CA1, CA1 region of the hippocampus; Cg, cingulate cortex; RSC, retrosplenial cortex.

**Figure 8:**
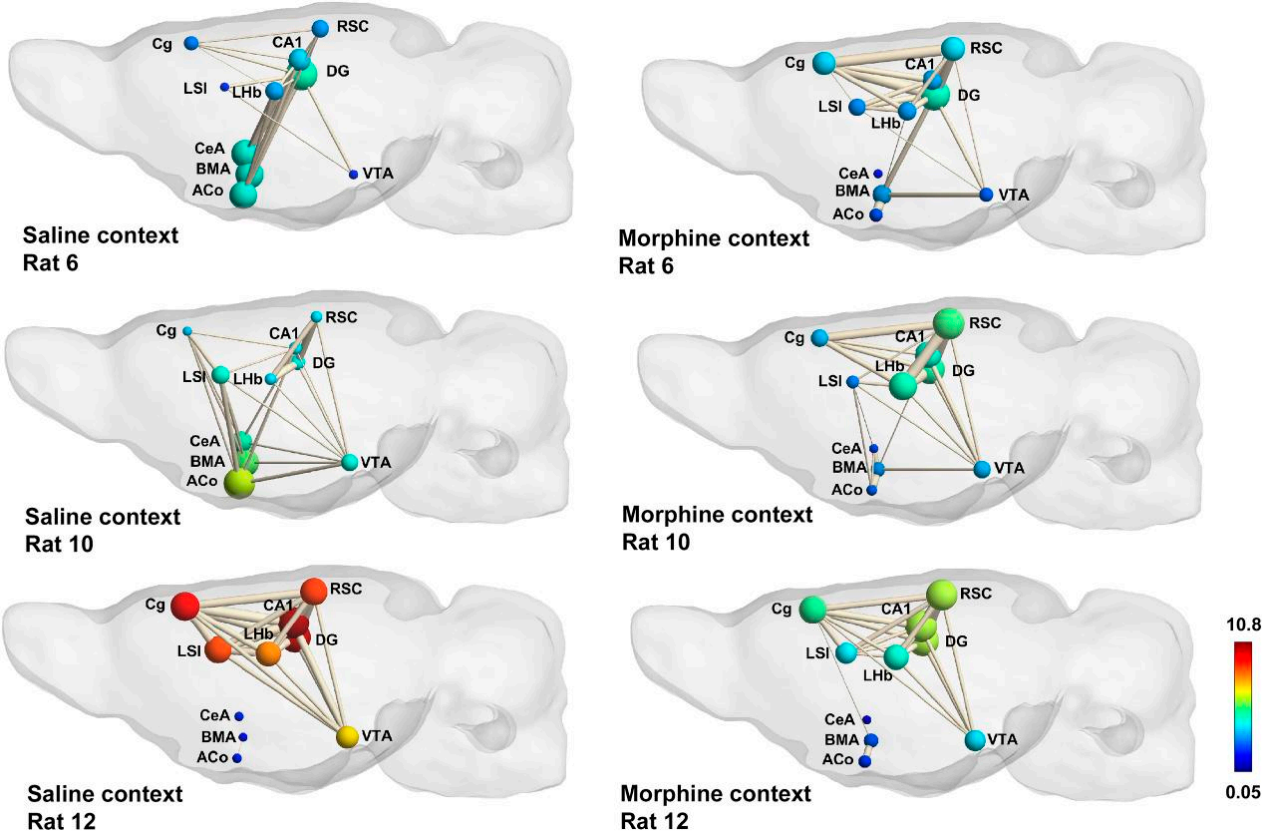
**Connectivity during saline context exposure is the better predictor of morphine-CPP test scoring—putative cortico-limbic network.** Graph theory representation of the putative cortico-limbic network during exposure to saline and morphine contexts for three border animals, two bad (rats 6 and 10) and one good learner (rat 12). Colour scale represents node colour, which was defined from a direct conversion of nodal degree into the jet coluor map code in MATLAB (scale limits were set based on the least and most central nodes in our analysis). Abbreviations: ACo, anterior cortical amygdaloid nucleus; LSI, lateral septum intermediate; LHb, lateral habenula; BMA, basomedial amygdala; CeA, central amygdala; Cg, cingulate cortex; VTA, ventral tegmental area; DG, dentate gyrus; CA1, CA1 region of the hippocampus; Cg, cingulate cortex; RSC, retrosplenial cortex.

We next tested whether connectivity during contextual cue presentation would correlate with performance in the CPP test. Using the same strategy, we plotted the *z*-scored connectivity upon saline- or morphine-associated cue presentation against individual CPP-deltas. Of the same 45 pairs of regions in the putative cortico-limbic network, 13 exhibited a significant correlation between connectivity on context presentation and CPP-delta. This was mainly found in the connections between amygdala, LSI and episodic memory regions on exposure to saline-associated cues (Fig. 7). Significant positive correlations were found in the LSI–LHb, LSI–RSC, VTA–CA1 and Cg–RSC pairs. Negative correlations were found in several ACo connections, namely, BMA, CeA, DG and RSC. Connectivity on exposure to morphine-associated cues was only correlated with CPP-delta in the LSI–Cg pair. Graph theoretical representations of the border animals are consistent with these findings, revealing high inter-individual variation in connectivity only on presentation of saline-associated contexts, where amygdala regions appear increasingly central in low CPP scoring rats, and memory-related regions, including the LSI, appearing highly central for the highest scoring rat (Fig. 8, left graphs).

CPP expression was uneven across animals, but the connectivity in response to morphine-associated cues in reward- and context-processing regions is similar, indicating that animals must have established a similar emotional association with the morphine-paired contexts. The connectivity in the same regions on exposure to saline-associated cues, on the other hand, is different among animals, indicating that different rats may have encoded different types of information across sessions of saline conditioning. In the saline-paired compartment, it is possible that a pre-anticipatory response to morphine administration and conditioning may have developed across sessions, stronger than the one developed during conditioning with morphine. It is also possible that a withdrawal/negative emotional state (due to absence of morphine) may have been encoded in the brain during saline conditioning. Our results align at the minimum with the first proposed explanation. As connectivity between ACo, a region implicated in odour-guided reward anticipation and other contextual (RSC and DG) and emotional-related (BMA) regions was higher in animals that did not develop CPP, it is likely that they also encoded saline-associated cues as rewarding. Such overlap may have hampered their ability to fully dissociate the two contexts’ relevance and rewarding value. This is further supported by the lower connectivity in salience assignment and contextual encoding regions such as the VTA, septum, hippocampus and cortex.

## Discussion

To investigate the brain-wide regional activity underlying drug addiction, we have developed a morphine-induced CPP protocol whose contextual cues can be consistently presented inside an MRI apparatus. Our fMRI data led to the identification of several brain structures classically attributed to functional neural circuits responsible for specific cognitive functions. We found widespread BOLD signal changes in response to morphine- and saline-paired context cue presentation, namely, regional circuitry involved in sensory processing (e.g. thalamus, somatosensory, piriform, visual and auditory cortices); action planning and initiation (dorsolateral striatum and motor cortex^48^); and spatial cognitive and emotional processing (e.g. cortical, anterior cingulate, insular, retrosplenial, parietal and temporal associative cortices; subcortical, nucleus accumbens, LSI, hypothalamus, amygdala, nucleus reuniens, ventral tegmental area, dorsal raphe and hippocampus).

Exposing rats to the sensory cues present in the distinct contexts led to BOLD responses in different regions according to context, suggesting that differentiation of context perception was achieved (Fig. 2C). In the presence of morphine-paired cues, we found activation of regions involved in the integration of limbic and mnemonic information (all PFC subdivisions, HIPP, DS, PRh, MEC and DLEnt),^49-51^ valence monitoring (BNST)^52^ and arousal (AHA, PHA, p1RF). This agrees with data from immediate early gene expression in PFC following response to cues associated to the opioid drug, heroin.^53^ The PFC (orbitofrontal, pre-limbic, infralimbic and cingulate cortices), along with the posterior cingulate cortex (PCC)—or RSC in rodents—is part of the DMN; a combination of cortical midline regions with enhanced activity during internally oriented cognitive processes, including mind wandering, imagination, future planning and memory retrieval. The pre-limbic and cingulate cortices have also been linked to the salience network (SN) in rodents. The SN is implicated in attribution of salience to environmental stimuli, visceral responses and interoceptive awareness and generally includes the anterior cingulate cortex, the amygdala, the nucleus accumbens and the anterior insular cortex.^54^ In healthy individuals, the SN directs attention to salient stimuli and allows, through means of the anterior insula, flexible and dynamic attentional shifts between internal (DMN-mediated) and external events, thereby enabling the disengagement from internal mental processes to goal-directed processes.^55^ In heroin-dependent individuals, disruptions to this dynamic inter-network connectivity have been observed, where the connectivity between the anterior insula (SN) and PCC (DMN) was shown to be increased.^56^ These disruptions are thought to underlie the enhanced attention towards drug-related cues and increased awareness towards internal states.^57^ In the rodent brain, the DMN is anchored in the RSC and the SN in the insular cortex. Optogenetic activation of anterior insular cortex neurons led to a decrease in this region's functional connectivity with the RSC, revealing how the salience network exerts control over the DMN in the rat brain.^58^ Tsai *et al*.^59^ showed an enhanced coupling of the SN and DMN in the rat brain during presentation of cues associated to heroin withdrawal, demonstrating a loss of control of the SN over the DMN and aligning with an attentional switch to internal emotional states, and possibly rumination, during withdrawal states.

Our brain activity maps seem to align with the previous findings, as the primary brain regions assigned to the DMN and SN showed an increased activity specifically in the presence of the morphine-paired cues. The increased activity co-occurring in signature regions of the two networks indicates that the rat brain has shifted its attention towards the saliency of the morphine-related cues. This is reflected, e.g. in the increased activity in the anterior insular cortex and Cg, and the internal interoceptive and emotional states that they predict.^60,61^ Within the insular cortex, we detected differences in the activity signal between its anterior and posterior subdivisions in response to morphine-related cues (Table 1), which is consistent with recent literature reporting functional heterogeneity within the insular cortex,^62^ with the anterior insula implicated in drug-seeking behaviour and real-time PP^63^ and the posterior insula in aversive sensory information-processing and affective states. The results from Fig. 7 provide empirical evidence that the individual responses are context-specific, rather than attributable to non-specific artefacts such as level of sedation. The medetomidine regimen used provides a level of sedation of a few hours for all animals, with temporally stable stimulus-evoked BOLD responses.^33^ By delivering the same dose to all animals at the same time points, and keeping the acquisition timeline under 1 h, a significant impact in BOLD signals is not anticipated.

In response to saline-paired cues, we detected changes in activity in regions involved in contextual^64^ and emotional memory,^65^ albeit distinct from the ones activated by morphine-paired cues: the medial septum (MS), vHIPP and periaqueductal grey (PAG) involved in avoidance of negative emotional states related to opioid withdrawal.^66,67^

Using network and seed-based connectivity analysis, we found that, after successive drug–context pairings, the LSI had increased its resting-state functional connectivity with the regions of a putative cortico-limbic network (Fig. 3C). Besides being the strongest non-HIPP target of HIPP CA3 outputs, LSI plays a central role in modulating motivated behaviour by integrating excitatory cortical inputs and regulating subcortical regions via tonic inhibition,^68^ namely, through GABA-mediated inhibition over VTA during encoding and recall of M-CPP.^69,70^ Enhanced LSI resting-state functional connectivity is consistent with a role in regulating the encoding of contextual cues associated to morphine reward. Whether these changes reflect compensatory mechanisms to counter a strong dopaminergic output from VTA, such as induced by morphine, or to facilitate VTA dopaminergic signalling in the HIPP remains an open question. Overall, the resting-state functional connections within our predefined cortico-limbic network did not strikingly change after animals underwent a regime of morphine administration and conditioning. However, the connection between the LHb and the CA1 region of the HIPP was strengthened (Fig. 3A). Though not directly connected anatomically, the LHb maintains coherent theta oscillatory activity with dorsal HIPP, directly related to memory performance in a spatial memory task.^71^ The LHb may, thus, play a role in encoding and consolidating contextual memories in the hippocampus, with either the drug or the contextual encoding, increasing synchrony. Another possible explanation for this increase in resting-state connectivity may be related to the encoding of saline-related contexts. The LHb is known to regulate negative emotional affect, namely, learning undesired experiences, and is also a critical component of an anti-reward system characterized by anticipation of aversive outcomes (in contrast with dopaminergic neuron activity) and a decrease in dopaminergic neuron firing in the VTA in response to failure to receive an expected reward.^72-74^ It is possible that, as a result of consistent alternations between drug and saline conditioning, LHb activity is highly dysregulated. For example, after a single administration of cocaine, Jhou *et al*.^75^ found biphasic LHb responses, with an initial activity decrease followed by increase, consistent with an initial rewarding, and subsequent aversive, effect. Notably, this increase in activity persisted for 7 days following the last injection in a self-administration protocol. Although in our study the LHb did not significantly increase centrality in the putative cortico-limbic network (*P* = 0.0629), we cannot discard the possibility that, at the time of fMRI acquisition, animals were already entering the first stages of withdrawal, as just 4 h prior they had visited the M-CPP apparatus without having received morphine. Altogether, the reinforced CA1–LHb connections and the largely consistent increase in LSI intra-network connectivity coincide with the processing, and likely consolidation, of emotionally charged episodic memories.

When morphine and saline-associated contexts are presented to the animals, a circuit formed by the amygdala’s cortical and subcortical divisions, the habenula and septum presents itself as a central hub for contextual discrimination (Fig. 4A). Connectivity changes between the anterior ACo, a region seemingly involved in odour-guided reward anticipation,^76^ and the LSI, via opposed signal correlated activity in morphine- and saline-paired contexts, are consistent with the LSI playing the role of a reward effect modulator (or lever), during the encoding of relevant contextual cues. The CeA and LHb also exhibit such opposed correlation dichotomy between morphine- and saline-paired contexts. We found anti-correlation during morphine context exposure and *vice versa* during saline context exposure. Ethanol addiction, binge-like intake of ethanol in mice, reveals enhanced activity of inhibitory projections from CeA to LHb.^77^ While we cannot ascertain the direction of this functional connection, and it is unclear whether negative correlations indeed reflect inhibitory processes at the cellular or circuit level,^4,78^ we consider plausible that the anti-correlation we found reflects the activity of such inhibitory synapse, resulting in silencing of anti-reward activity in LHb.

The level of functional connectivity between LHb and the three subdivisions of the amygdala, assessed during pre-CPP resting-state fMRI, predicts individual M-CPP behaviour (Fig. 5). In rats with low CPP-delta, we observed negative or near-zero correlation coefficients between these regions, while higher-scoring rats show positive coefficients. We did not find any correlation between CPP-delta scores and the connectivity within memory regions. Such correlations were specific to regions implicated in regulating negative affect, leading us to speculate that they reflect distinct circuit organization or inhibitory/excitatory connection imbalances among these regions across different animals. Furthermore, we find that connectivity levels within our limbic network were highly predictive of M-CPP scores during exposure to saline-associated cues (Fig. 7), perhaps reflecting a parallel between the strength of CPP memory and the intensity of withdrawal-related neural circuit activity. The fact that, on exposure to morphine-paired cues, the levels of connectivity across regions involved in memory, reward and affect are not predictive of observed M-CPP-deltas implies that neural functional connectivity is similar during morphine conditioning, and the differentiator factor might consist in either neural plasticity mechanisms at work during saline conditioning (S-CPP), as they preceded morphine conditioning in the same day, or the processing of short-intermittent withdrawal states throughout conditioning sessions. Further exploration is needed to understand whether low-scoring rats were protected from developing addiction due to being unable to encode or segregate different types of information in dedicated neural assemblies, or exhibited an intensified negative response to withdrawal that overshadowed their memory of morphine reward in the same context, or their motivation to approach the previously morphine-paired context. Alternatively, they may have established an aversive association with morphine administration and conditioning, attributing saline conditioning (absence of morphine) a rewarding value. We tested the hypothesis that low-scoring rats showed a higher functional connectivity among stress- and aversion-related regions during presentation of morphine-paired cues. We found no correlation between bed nucleus of the stria terminalis (BNST), posterior insular cortex and the lateral habenula, all related to stress and aversive states,^79-81^ with CPP performance (data not shown), which strongly indicates that this behaviour is not likely to result from an aversive reaction to morphine.

Even though classical CPP induced a robust conditioning (Supplementary Fig. 1C), the fMRI-adapted CPP resulted in more inter-individual variability in scores. It is difficult to compare this data with other published results on morphine-CPP, due to the fMRI requirement of a simplified version of both the conditioning sequence and contexts, in opposition to the wide visual and tactile cues available to the classical CPP apparatuses. Furthermore, in rats under the same morphine regimen, the vulnerability to develop CPP is predicted by a delayed activity in novel environments^82^ which indicates that underlying individual behavioural factors are also involved. Our data provides naive brain functional connectivity-based evidence, with lower levels of CPP being associated to how the lateral habenula and amygdala are functionally connected.

We found a common circuitry supporting the neural mechanisms responsible for memorizing an association between distinct contexts, and morphine or the absence thereof that includes regions involved in affective, reward, contextual perception and memory. Individual circuit mapping was associated to distinct addictive outcomes. Animals exhibiting stronger emotional responses (as measured by the baseline resting-state amygdala and habenula functional connectivity) display a neural circuit priming effect, resulting in the development of stronger connectivity patterns, therefore anticipating stronger morphine addiction behaviour. These findings may clarify the inter-individual sensitivity and vulnerability seen in addiction to opioids found in humans.

## Supplementary Material

fcae281_Supplementary_Data

## Data Availability

All the software used for data analysis and the respective information is provided in each respective section. The data that support the findings of this study are available from the corresponding authors upon reasonable request. The activity analysis code in Python and the connectivity analysis MATLAB scripts are available in GitHub (https://github.com/joana-g-ribeiro/rat-addiction-fMRI-).
