# Supplementary material for: Mapping functional traces of opioid memories in the rat brain
Source: Brain Commun. 2024 Aug 19;6(5):fcae281. doi: 10.1093/braincomms/fcae281 (PMC11369824; doi:10.1093/braincomms/fcae281)

Supplementary Table I. Mean BOLD signal changes in response to saline and morphine-paired cues in the last 30 sec of stimuli presentation (at the end of the 10-min blocks) in the whole brain. Up and down arrows indicate positive and negative BOLD signal changes, respectively. Anatomical identification of activated clusters was achieved by superimposing Paxinos and Watson (2007)* brain atlas figures onto the activity maps of Supplementary Figure 2.

Brain regions	BOLD signal change	Morphine cue-specific	Saline cue-specific	Common to morphine and saline cues
**Olfactory nuclei**	↑			Anterior olfactory nuclei (dorsal and medial subdivisions)
	↓			
**Prefrontal cortex**	↑			Orbitofrontal (ventral and lateral subdivisions) cortex
	↓			
**Basal forebrain**	↑	Ventral pallidum, Endopiriform nucleus, Anterior cortical amygdaloid nucleus (ACo), Anterior amygdaloid area, Basomedial amygdala (BMA)	Nucleus accumbens (core)	Claustrum, Dorsal striatum
	↓		Dorsal striatum (left hemisphere), Globus pallidus, Central amygdala (CeA), Basolateral amygdala (BLA), Lateral amygdaloid nucleus	Ventral claustrum
**Hypothalamus**	↑			
	↓	Posterior hypothalamic nucleus, Supramammillary nucleus		
**Cerebral cortex**	↑	Anterior insular, Primary motor, Parietal association (lateral), and Primary visual cortices	Secondary motor, Cingulate, Retrosplenial, and Ectorhinal cortices	
	↓	Somatosensory, Motor (posterior segment), Parietal association (medial), Retrosplenial, and Secondary visual cortices		Posterior insular, Primary and Secondary auditory, and Entorhinal (dorsolateral) cortices
**Hippocampus**	↑	Dorsal CA1	Dorsal DG	Dorsal subiculum
	↓		Ventral CA1	

**Thalamus**	↑		Medial pretectal nucleus, Lateral posterior thalamic nucleus	
	↓			
**Midbrain**	↑		Periaqueductal gray (dorsomedial subdivision)	Superior colliculus
	↓	Ventral tegmental area		

Supplementary Table II. Differences in mean BOLD signal between saline and morphine-paired cue responses in the last 30 sec of stimuli presentation (at the end of the 10-min blocks) in the whole brain. Activated clusters in ‘saline cue’ condition were subtracted from ‘morphine cue’ condition, and vice-versa, to obtain magnitude difference maps. Anatomical identification of activated clusters was achieved by superimposing Paxinos and Watson (2007)* brain atlas figures onto the activity maps of Supplementary Figure 2.

**Brain regions**	**Difference morphine - saline cues**	**Difference saline -morphine cues**
**Basal forebrain**	Anterior cortical amygdaloid nucleus (ACo), Magnocellular preoptic nucleus (MCPO), Medial amygdaloid nucleus (Me)	
**Cerebral cortex**		Primary and secondary motor, Parietal association, and Retrosplenial cortices
**Thalamus**		Lateral habenula
**Midbrain**		Superior colliculus

*Reference:
Paxinos, G. and Watson, C. (2007) The Rat Brain in Stereotaxic Coordinates. 6th Edition, Academic Press, San Diego.

Supplementary Figure 1 – Validation of the contextual cues for fMRI adaptation of the CPP task.

a. Spontaneous preference to indicated odorants relative to water (analysis of variance with post-hoc tests (Fisher LSD) performed on normalized investigation times). Values presented as mean ± SEM. *P < 0.05; **P < 0.01. n = 8 rats.
b. Permanence times in compartments during a 15-min free exploration of the CPP apparatus under exposure to single odorants (i) and (ii); white light LEDs (array and single light point) (iii); and the combination of the best group of odorants and visual stimuli (iv). All values are presented as mean ± SEM. *P < 0.05; **P < 0.01; ***P < 0,001 (Two-way ANOVA followed by Bonferroni's multiple comparisons test). n = 10 rats.
c. i. Rats developed preference for the morphine-paired context, as revealed by the time spent in the morphine-paired compartment relative to time spent in the saline-paired compartment at post-conditioning. A two-way repeated measures ANOVA followed by Bonferroni's multiple comparisons test was employed to analyze differences between times spent in the two chambers. Values are presented as mean ± SEM. **P < 0.01. ii. Individual permanence times in the two CPP compartments in the pre- and post-conditioning sessions shows that M-CPP was expressed in most animals of the group (8 out of 10). n = 10 rats.

Supplementary Figure 2 – Responses to saline- and morphine-associated contexts by the end of stimulus presentation.

Representative anatomical scans (T2-weighted anatomical MRI image of our template brain) display: i. and ii.: Mean BOLD activity maps in the second-to-last 30-s window in response to the morphine- and saline-associated cues, respectively. Color scale represents mean regression coefficients for morphine and saline contrasts in comparison to baseline (masked under a significance level set at 0.05 and a cluster size threshold of 16 voxels); and iii. and iv: Magnitude maps show the clusters that survived the difference between activated clusters during presentation of saline and morphine cues, and vice-versa. Color scale represents P values of the surviving voxels. Coordinates under each brain section represent Paxinos & Watson's rat brain atlas coordinates (in mm). n = 12 rats.

Supplementary Figure 3 – Resting state connectivity based on the seed-based analysis of the putative cortico-limbic network.

Representative anatomical scans (T2-weighted anatomical MRI image of our template brain) display mean statistical t maps of resting state connectivity for the five supplementary regions within our putative cortico-limbic network, converted into seeds for seed-based analysis. Color scale represents t-value i.e., strength of functional connectivity of the seed region with all voxels in the brain. Abbreviations: ACo = anterior cortical amygdaloid nucleus, BMA = basomedial amygdala, CeA = central amygdala, VTA = ventral tegmental area, Cg = cingulate cortex. Coordinates under each brain section represent Paxinos & Watson's rat brain atlas coordinates (in mm). n = 12 rats.

Supplementary Figure 4 – Functional connectivity in pre- and post-CPP, under saline and morphine context exposure for sensory and limbic regions.

a. Functional connectivity (FC) between pre- and post-CPP in an olfactory-limbic-memory network. i. Upper panel: Mean FC-matrices of pre-CPP (lower half) and post-

CPP (upper half) contexts. X and Y-axes represent brain regions. Color scale represents Pearson correlations i.e., strength of FC between each pair of brain regions. ii. FDR-adjusted P value matrix representing statistically significant connectivity differences between pre- versus post-CPP. Color scale represents P values (0-0.05). X- and Y-axes represent brain regions. n = 12 rats.

b. Functional connectivity (FC) between saline- and morphine-paired contexts in an olfactory-limbic-memory network. i. Upper panel: Mean FC-matrices of saline (lower half) and morphine (upper half) contexts. X and Y-axes represent brain regions. Color scale represents Pearson correlations i.e., strength of FC between each pair of brain regions. ii. FDR-adjusted P value matrix representing statistically significant connectivity differences between saline versus morphine. Color scale represents p values (0-0.05). X- and Y-axes represent brain regions. n = 12 rats.
c. FDR-adjusted P value matrices representing statistically significant connectivity differences between i. morphine versus post-CPP and ii. saline versus post-CPP. Color scale represents p values (0-0.05). X- and Y-axes represent brain regions. Abbreviations: Pir = piriform cortex, AON = anterior olfactory nucleus, LOT = nucleus of the lateral olfactory tract, Tu = olfactory tubercle, CeA = central amygdala, ACo = anterior cortical amygdaloid nucleus, CA1 = CA1 region of the hippocampus, DG = dentate gyrus, DLEnt = dorsolateral entorhinal cortex, MEC = medial entorhinal cortex. n = 12 rats.
d. Functional connectivity (FC) between pre- and post-CPP in an visual-limbic-memory network. i. Upper panel: Mean FC-matrices of pre-CPP (lower half) and post-CPP (upper half) contexts. X and Y-axes represent brain regions. Color scale represents Pearson correlations i.e., strength of FC between each pair of brain regions. ii. FDR-adjusted P value matrix representing statistically significant connectivity differences between pre- versus post-CPP. Color scale represents P values (0-0.05). X- and Y-axes represent brain regions. n = 12 rats.
e. Functional connectivity (FC) between saline- and morphine-paired contexts in an olfactory-limbic-memory network. i. Upper panel: Mean FC-matrices of saline (lower half) and morphine (upper half) contexts. X and Y-axes represent brain regions. Color scale represents Pearson correlations i.e., strength of FC between each pair of brain regions. ii. FDR-adjusted P value matrix representing statistically significant connectivity differences between saline versus morphine. Color scale represents p values (0-0.05). X- and Y-axes represent brain regions. n = 12 rats.
f. FDR-adjusted P value matrices representing statistically significant connectivity differences between i. morphine versus post-CPP and ii. saline versus post-CPP. Color scale represents p values (0-0.05). X- and Y-axes represent brain regions. Abbreviations: LGN = lateral geniculate nucleus, V1 = primary visual cortex, InG = intermediate gray layer of the superior colliculus, SC = superior colliculus, CeA = central amygdala, ACo = anterior cortical amygdaloid nucleus, CA1 = CA1 region of the hippocampus, DG = dentate gyrus, DLEnt = dorsolateral entorhinal cortex, MEC = medial entorhinal cortex. n = 12 rats.

Supplementary Figure 1 - Validation of the contextual cues for fMRI adaptation of the CPP test

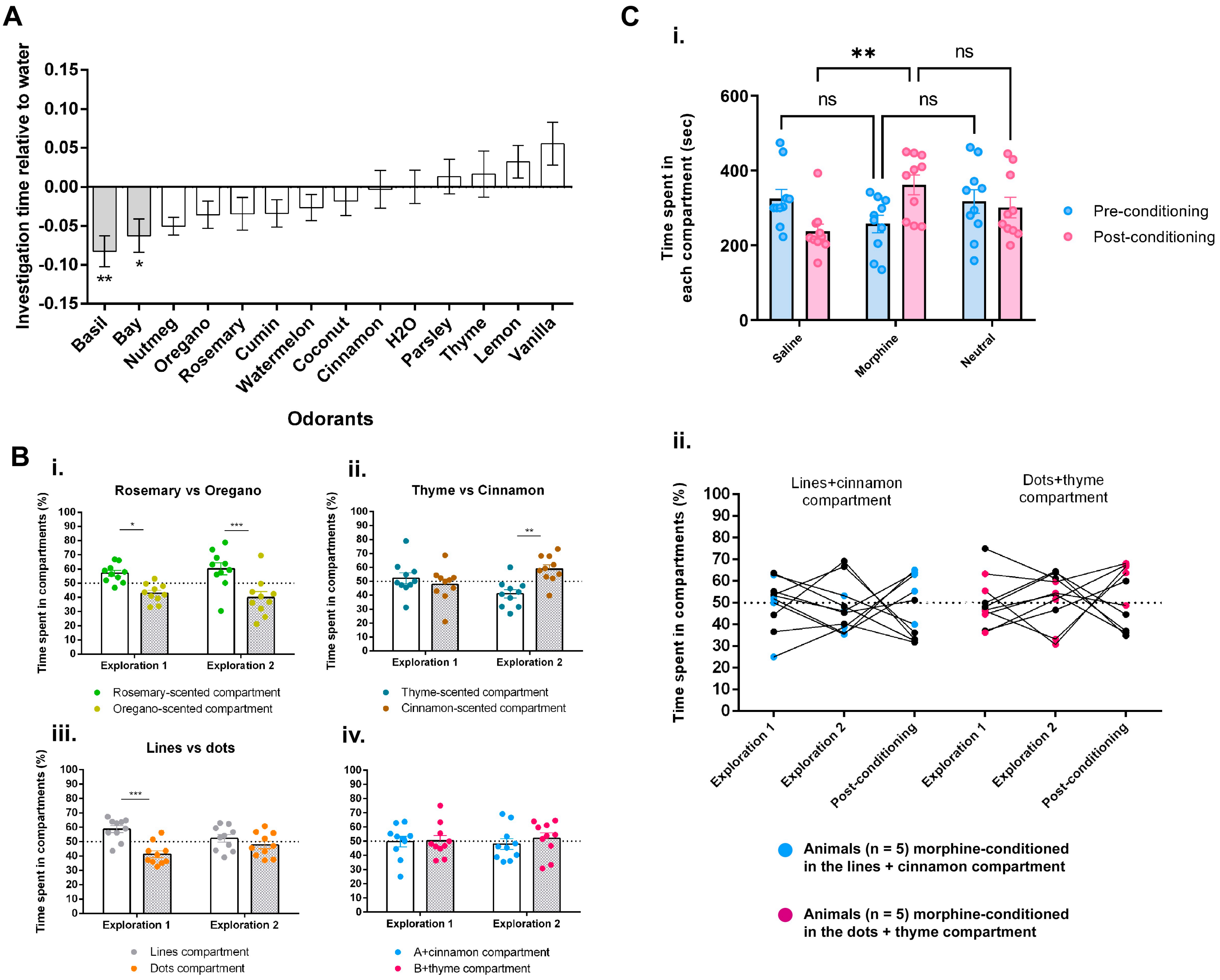

Supplementary Figure 2 - Responses to saline- and morphine-associated cues by the end of stimulus presentation

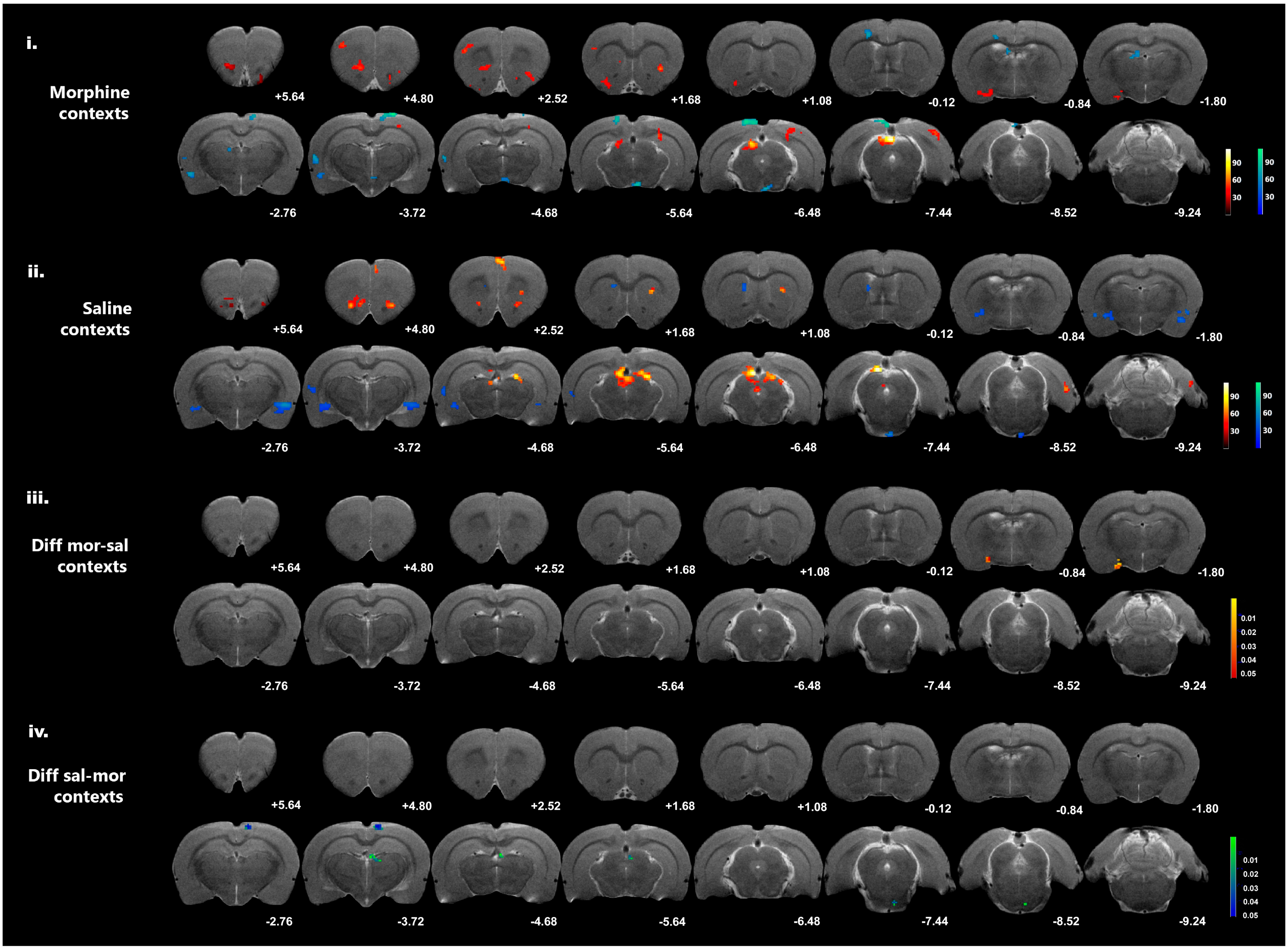

Supplementary Figure 3 - Resting state connectivity based on the seed-based analysis of the putative cortico-limbic network

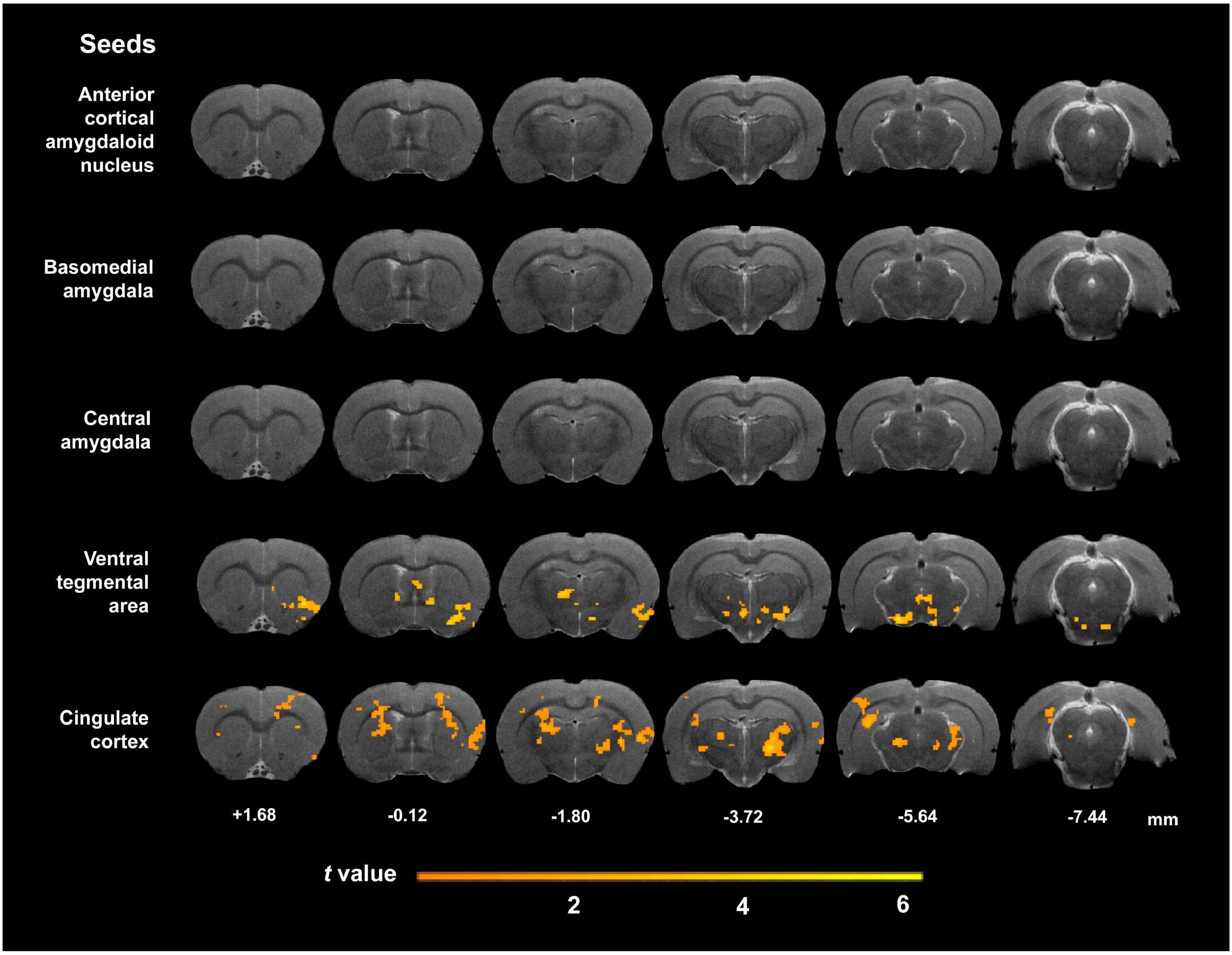

Supplementary Figure 4 - Connectivity in sensory regions

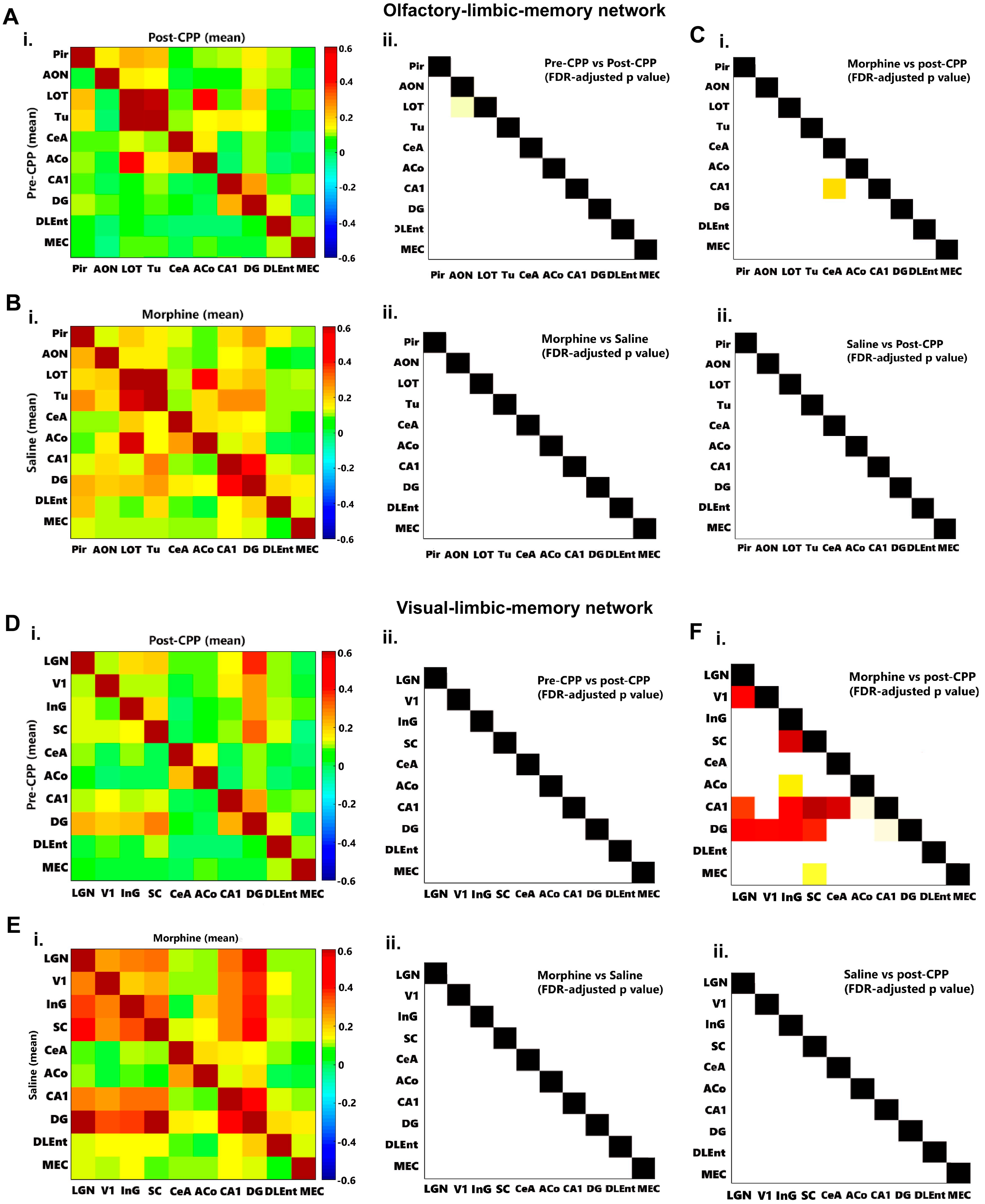

Supplement: fcae281_Supplementary_Data [file fcae281_supplementary_data.pdf]
